# Genome Scale Analysis Reveals IscR Directly and Indirectly Regulates Virulence Factor Genes in Pathogenic *Yersinia*

**DOI:** 10.1128/mBio.00633-21

**Published:** 2021-06-01

**Authors:** David Balderas, Erin Mettert, Hanh N. Lam, Rajdeep Banerjee, Tomas Gverzdys, Pablo Alvarez, Geetha Saarunya, Natasha Tanner, Adam Zoubedi, Yahan Wei, Patricia J. Kiley, Victoria Auerbuch

**Affiliations:** a Department of Microbiology and Environmental Toxicology, University of California, Santa Cruz, Santa Cruz, California, USA; b Department of Biomolecular Chemistry, University of Wisconsin—Madison, Madison, Wisconsin, USA; c Department of Biological Sciences, University of South Carolina, Columbia, South Carolina, USA; National Institute of Child Health and Human Development (NICHD)

**Keywords:** *Yersinia*, *Yersinia pseudotuberculosis*, ChIP-Seq, IscR, RNA-Seq, *Yersinia pestis*

## Abstract

The iron-sulfur cluster coordinating transcription factor IscR is important for the virulence of Yersinia pseudotuberculosis and a number of other bacterial pathogens. However, the IscR regulon has not yet been defined in any organism. To determine the *Yersinia* IscR regulon and identify IscR-dependent functions important for virulence, we employed chromatin immunoprecipitation sequencing (ChIP-Seq) and RNA sequencing (RNA-Seq) of Y. pseudotuberculosis expressing or lacking *iscR* following iron starvation conditions, such as those encountered during infection. We found that IscR binds to the promoters of genes involved in iron homeostasis, reactive oxygen species metabolism, and cell envelope remodeling and regulates expression of these genes in response to iron depletion. Consistent with our previous work, we also found that IscR binds *in vivo* to the promoter of the Ysc type III secretion system (T3SS) master regulator LcrF, leading to regulation of T3SS genes. Interestingly, comparative genomic analysis suggested over 93% of IscR binding sites were conserved between Y. pseudotuberculosis and the related plague agent Yersinia pestis. Surprisingly, we found that the IscR positively regulated *sufABCDSE* Fe-S cluster biogenesis pathway was required for T3SS activity. These data suggest that IscR regulates the T3SS in *Yersinia* through maturation of an Fe-S cluster protein critical for type III secretion, in addition to its known role in activating T3SS genes through LcrF. Altogether, our study shows that iron starvation triggers IscR to coregulate multiple, distinct pathways relevant to promoting bacterial survival during infection.

## INTRODUCTION

Iron is an important cofactor for many proteins involved in respiration, oxidative stress resistance, gene regulation, and other processes (1). Most bacteria require ∼10^−6^ M iron to support optimal growth. Yet, in the mammalian host, the level of free iron is only 10^−18^ M due to the concerted action of mammalian iron storage and carrier proteins (2, 3). During infection, the amount of available iron decreases even further as a result of nutritional immunity, a process through which inflammatory mediators lead to further sequestration of iron (4). Yersinia pseudotuberculosis and the related enteropathogen Yersinia enterocolitica cause self-limiting mesenteric lymphadenitis and gastroenteritis in immunocompetent people as well as serious disseminated infection in iron-overloaded individuals (5, 6). The plague agent Yersinia pestis is closely related to Y. pseudotuberculosis, emerging as a distinct species ∼1,500 to 6,400 years ago (7). The elevated susceptibility of iron-overloaded individuals to disseminated *Yersinia* infection underscores the low iron bioavailability experienced by *Yersinia* in mammalian tissues other than the intestinal lumen, despite expression of multiple iron uptake systems (8–10). *In vitro*, enteropathogenic *Yersinia* can utilize both inorganic and heme iron sources through a number of iron uptake systems typical of many pathogens (11–14). Although the importance of iron availability in *Yersinia* infection is well accepted, the transcription regulatory networks that operate under iron starvation conditions have not been firmly established.

In many pathogenic bacteria, expression of iron uptake systems and virulence factors is controlled by the conserved global iron regulator Fur (15). Although multiple iron uptake systems are controlled by Fur in *Yersinia*, including yersiniabactin, the key siderophore in pathogenic *Yersinia*, Fur has not been shown to directly regulate known virulence factors other than those involved in metal acquisition (16, 17). Rather, we discovered that expression of a key virulence factor, the *Yersinia* secretion (Ysc) type III secretion system (T3SS), encoded on a 70-kb virulence plasmid pYV/pCD1 (18), was controlled by a different iron-regulated transcription factor, IscR (9, 19). Subsequently, IscR has been shown to coordinate virulence factor expression in multiple pathogens (20–24).

In Escherichia coli where IscR was discovered, DNA binding is regulated by ligation of an Fe-S cluster (25), providing a linkage between iron availability and activity of this transcription factor. E. coli IscR controls the expression of more than 40 genes, including anaerobic metabolism and respiration, and the Isc and Suf Fe-S cofactor biogenesis systems (26). IscR exists in either a holo form, when it is bound to a [2Fe-2S] cluster, or in an apo form, which is clusterless (25). While holo-IscR binds to so-called type I motif sequences with a significantly higher affinity than apo-IscR, both holo-IscR and apo-IscR can bind to type II motif sequences (25, 27, 28). IscR can both activate and repress transcription depending on the position of its binding site relative to a promoter. Bioavailability of iron, oxygen tension, and reactive oxygen species have all been inferred to affect the relative ratio of holo- to apo-IscR (25, 29–35). In turn, holo-IscR negatively regulates *iscR* transcription through two type I motif sequences in the *isc* operon promoter, leading to an increase in overall IscR levels under aerobic and/or iron-starved conditions (9, 25), and derepression of the Isc and Suf biogenesis pathways to maintain Fe-S cluster homeostasis (25, 26).

In *Yersinia*, IscR binds to a type II motif in the promoter of the gene encoding LcrF, the master regulator of the Ysc T3SS (9, 19). IscR, and subsequently LcrF, levels increase with oxygen tension through derepression of the *isc* operon, driving T3SS expression (9). While the T3SS is important for *Yersinia* virulence, Y. pseudotuberculosis lacking T3SS expression can still colonize the intestinal lumen as well as the mesenteric lymph nodes (9, 36–38). In contrast, Y. pseudotuberculosis lacking *iscR* is defective in colonization of all mouse tissues tested (9, 19). This suggests that IscR regulates additional virulence factors in *Yersinia.* In order to assess further how IscR contributes to *Yersinia* virulence, we used whole transcriptome RNA sequencing (RNA-Seq) and chromatin immunoprecipitation with massively parallel DNA sequencing (ChIP-Seq) to identify genes directly regulated by IscR following iron starvation, which *Yersinia* experiences during disseminated infection. Interestingly, comparative genomics revealed very high predicted conservation of the IscR regulon in Y. pestis. One highly conserved IscR binding site was found in the promoter of the *suf* Fe-S cluster biogenesis operon. Surprisingly, our data show that IscR direct regulation of the Y. pseudotuberculosis *suf* operon is critical for T3SS activity under iron-depleted conditions.

## RESULTS

### Identification of IscR and iron-regulated genes in Y. pseudotuberculosis.

**FIG 1:**
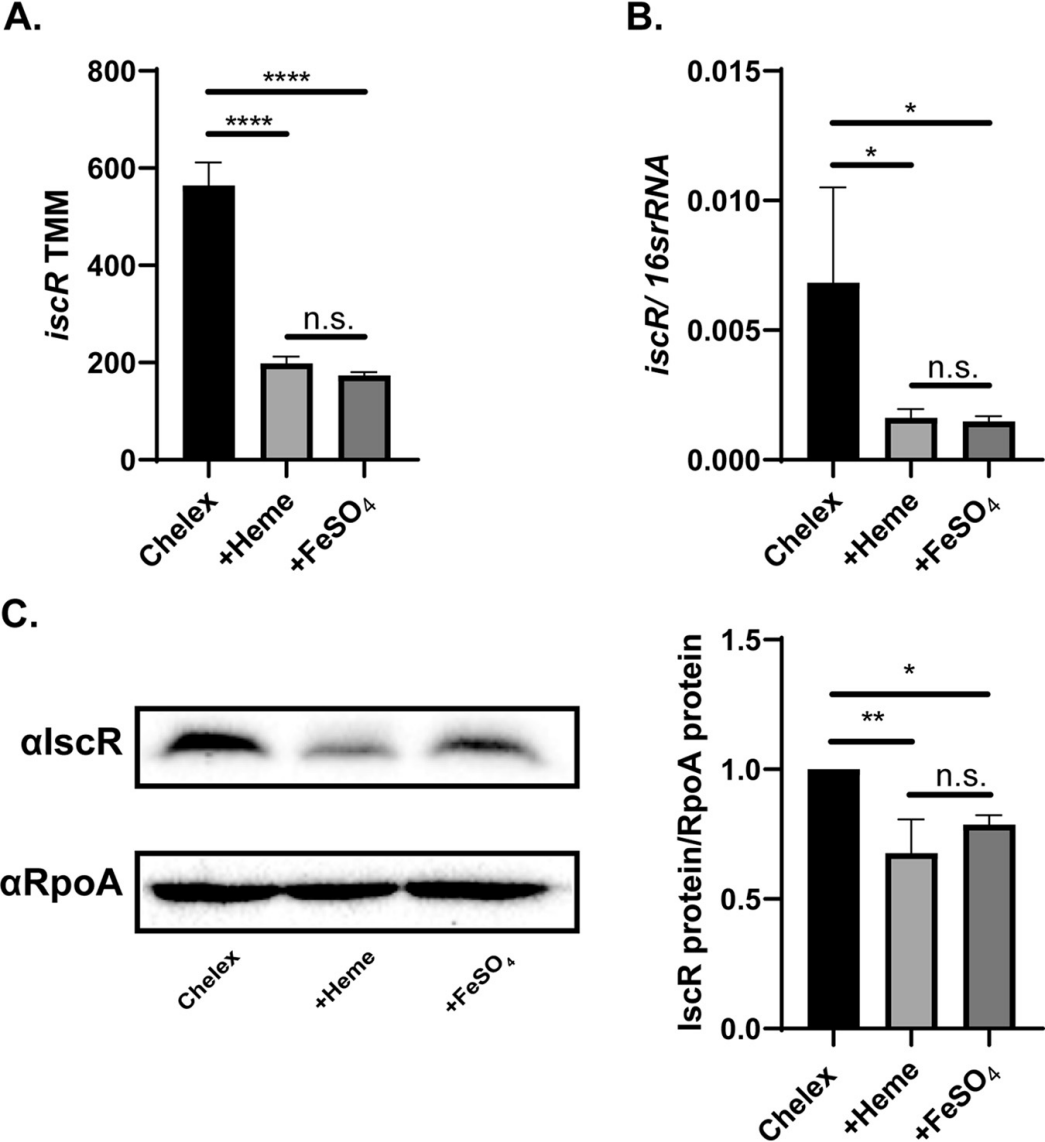
IscR mRNA and protein levels decrease upon iron supplementation. (A) Expression of *iscR* in Y. pseudotuberculosis under various iron conditions, as measured by RNA-Seq. Reads are represented by trimmed mean of M-values (TMM) of WT in Chelex-treated M9 minimal medium with no iron source added back (Chelex), supplemented with 5 μM hemin (+Heme), or supplemented with 3.6 μM FeSO_4_ (+FeSO_4_). ****, *P* < 0.0001; n.s., not significant (EdgeR with a corrected FDR *post hoc* test). (B) Expression of *iscR* mRNA relative to 16S rRNA, as measured by quantitative reverse transcription-PCR (qRT-PCR). *, *P* < 0.05 (one-way ANOVA with Dunnett’s *post hoc* test). (C) IscR protein levels in whole-cell extracts from WT Y. pseudotuberculosis, as detected using an anti-IscR antibody (αIscR). RpoA served as a loading control. Shown is the average of three biological replicates ± standard deviation. **, *P* < 0.01; *, *P* < 0.05 (one-way ANOVA with Dunnett’s *post hoc* test).

*Yersinia* experience iron starvation during extraintestinal infection (39, 40). Since IscR levels and activity are regulated by iron, we chose to assess the bacterial transcriptional response following iron starvation. Wild-type (WT) and Δ*iscR* Y. pseudotuberculosis bacteria were starved for iron using previously published methods (see Materials and Methods) (12). Cultures either remained iron limited (no iron added back to Chelex-treated media) or were supplemented with organic iron (5 μM hemin) or inorganic iron (3.6 μM FeSO_4_), which can both be utilized by *Yersinia* via distinct uptake systems (11–14), and incubated for 3 h at 37°C. RNA-Seq analysis showed that levels of IscR mRNA and protein were highest following prolonged iron starvation compared to after iron supplementation (Fig. 1A to C). As holo-IscR negatively regulates *isc* operon transcription, these data are consistent with a decrease in holo-IscR activity following iron starvation and suggest that these growth conditions modulate IscR-regulated gene expression and provide a mimic of conditions found in the host. Indeed, we previously found that even adding back only 0.036 μM FeSO_4_ prevented derepression of *iscR* expression (9).

**FIG 2:**
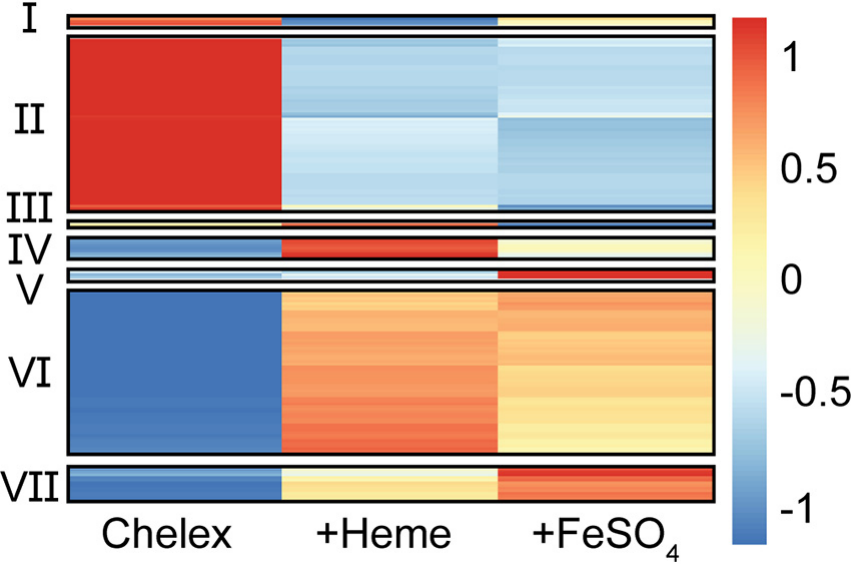
Iron supplementation modulates the expression of 1,373 genes in *Yersinia.* RNA-Seq was carried out in WT Y. pseudotuberculosis after iron starvation (Chelex) or followed by supplementation with heme or FeSO_4_, as in Fig. 1. Cluster analysis was performed on the average RNA-Seq reads normalized by trimmed mean of M-values (TMM) for all 1,373 differentially expressed genes. Expression values were transformed and scaled by gene before cluster analysis. The color bar shows relative gene expression.

Global RNA-Seq analysis revealed a number of genes that respond to iron availability. We used clustering analysis to sort these differentially expressed genes into seven groups based on the relative changes in expression following prolonged iron starvation compared to supplementation with inorganic iron or heme. The two largest clusters were genes that were either downregulated (cluster VI) or upregulated (cluster II) in response to iron limitation. (Fig. 2; see Data Set S1 in the supplemental material). Cluster VI and II genes also showed similar expression between the two iron sources. Cluster II genes contained *iscR* in addition to genes involved in iron uptake (ex-*hemPR*, *hmuSTUV*, *iutA/iucABCD*, and *feoB*), iron sulfur cluster biogenesis (*sufABCDSE*), lipopolysaccharide (LPS) biosynthesis and modification (*arnABCDE*, *lpxABDTL*, *rfaQ*, and *pagP*), lipoprotein and outer membrane protein targeting (*lolCDE*, *bamA*, *surA*, and *skp*), cell wall remodeling and defense, the *dusB*-*fis* virulence-associated operon, and the T3SS. Upregulation of iron uptake genes and the *suf* operon in response to iron starvation was expected given previous studies (12, 14, 33), but regulation of LPS modification, periplasmic protein targeting, and cell wall remodeling by iron has not been previously reported in *Yersinia*. Cluster VI included genes involved in energy metabolism (*sucABCD*, *fdoGHI*, and *dmsAD*), antioxidants (*sodB*, *katAG*, and *ompW*), and the AI-2 autoinducer pathway (*lsrR* regulator and *lsr* operon). Altogether, these results suggest that Y. pseudotuberculosis increases iron metabolism and cell envelope remodeling during iron starvation, a condition known to be experienced by *Yersinia* during extraintestinal infection (39, 40).

10.1128/mBio.00633-21.8 DATA SET S1 Transcriptomic data of the Y. pseudotuberculosis wild type and the Δ*iscR* mutant under both iron-starved and non-iron-starved conditions. Download Data Set S1, XLSX file, 3.6 MB. Copyright © 2021 Balderas et al. 2021 Balderas et al. https://creativecommons.org/licenses/by/4.0/ This content is distributed under the terms of the Creative Commons Attribution 4.0 International license.

**FIG 3:**
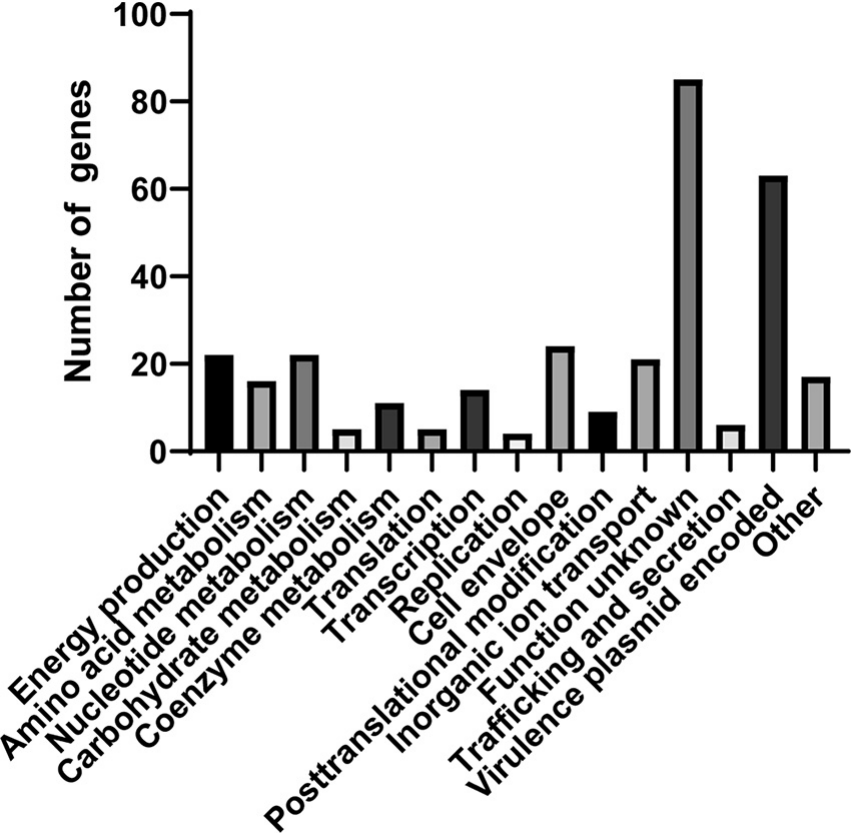
Deletion of *iscR* leads to expression changes in genes involved in virulence, ion transport, cell envelope, and other processes following prolonged iron starvation. Clusters of Orthologous Groups of proteins (COG) analysis of genes differentially expressed between the WT and Δ*iscR* strains following iron starvation.

**FIG 4:**
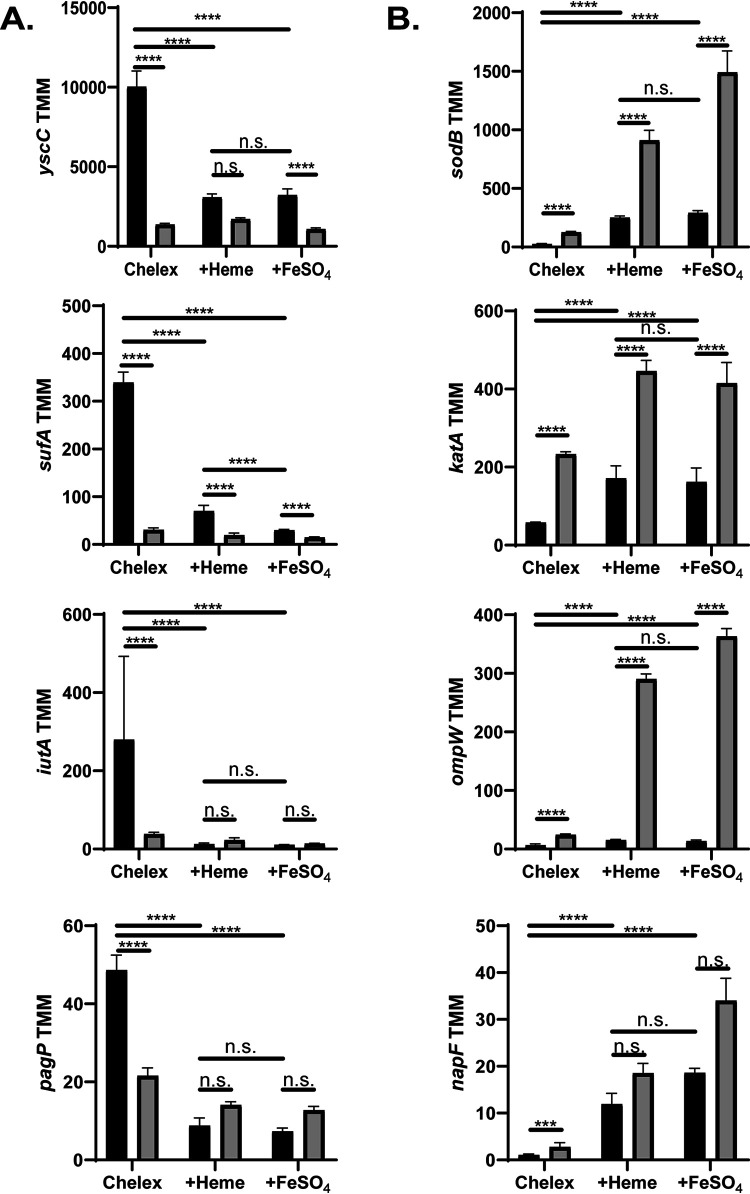
Several *Yersinia* genes follow expression patterns consistent with activation or repression by apo-IscR. Expression of various genes as measured by RNA-Seq. Reads are represented by trimmed mean of M-values (TMM) of WT (black) and Δ*iscR* strains (gray). ****, *P* < 0.0001; n.s. not significant (EdgeR with a corrected FDR *post hoc* test). (A) Genes predicted to be activated by apo-IscR. (B) Genes predicted to be repressed by apo-IscR.

Genes that are differentially regulated in response to changes in iron availability may be controlled by IscR, Fur, and possibly other transcription factors or may be indirectly regulated by these factors. In order to determine which genes are affected by IscR, we carried out the same RNA-Seq analysis as above with the Δ*iscR* mutant. Under prolonged iron starvation (Chelex condition), when IscR levels are highest in the WT strain, a total of 324 genes were differentially expressed in the wild-type strain versus the Δ*iscR* strain (Fig. 3; Data Set S1). Of the 127 genes whose expression was greater in the wild type than in the Δ*iscR* mutant, 48 (∼38%) were carried on pYV and were among the genes with the largest fold changes between the wild-type and Δ*iscR* strains. As our previous data showed that apo-IscR directly regulates the T3SS master regulator LcrF, the majority of these pYV-encoded genes are likely to be indirectly regulated by IscR. In iron-limited conditions, such as those encountered by *Yersinia* during extraintestinal infection, the dominant form of IscR is predicted to be apo-IscR. Genes induced by apo-IscR would be expected to be more highly expressed during iron starvation compared to after iron supplementation as well as have decreased expression upon deletion of *iscR*. Genes whose expression patterns were consistent with apo-IscR induction of their promoters included the T3SS genes *yscCDEFGHK*, the *sufABCDSE* operon, metal transport genes *iucABCDiutA*, *alcC*, *cirA*, *fhuC_1*, and *oprC*, and cell envelope biosynthesis and remodeling genes *pagP*, *amiD*, and *ydhO* (Fig. 4A and Data Set S1). As expected, the 197 genes expressed at lower levels in the wild type compared to the Δ*iscR* strain under prolonged iron starvation included those in the *isc* operon. Genes repressed by apo-IscR would be expected to have higher mRNA levels following iron supplementation compared to iron starvation in wild-type bacteria as well as have increased expression upon deletion of *iscR*. Genes whose expression patterns are consistent with apo-IscR repression include *sodB*, *katA*, *ompW*, and the [4Fe-4S] cluster protein *napF* (Fig. 4B).

### ChIP-Seq analysis of *in vivo* IscR binding in Y. pseudotuberculosis.

**FIG 5:**
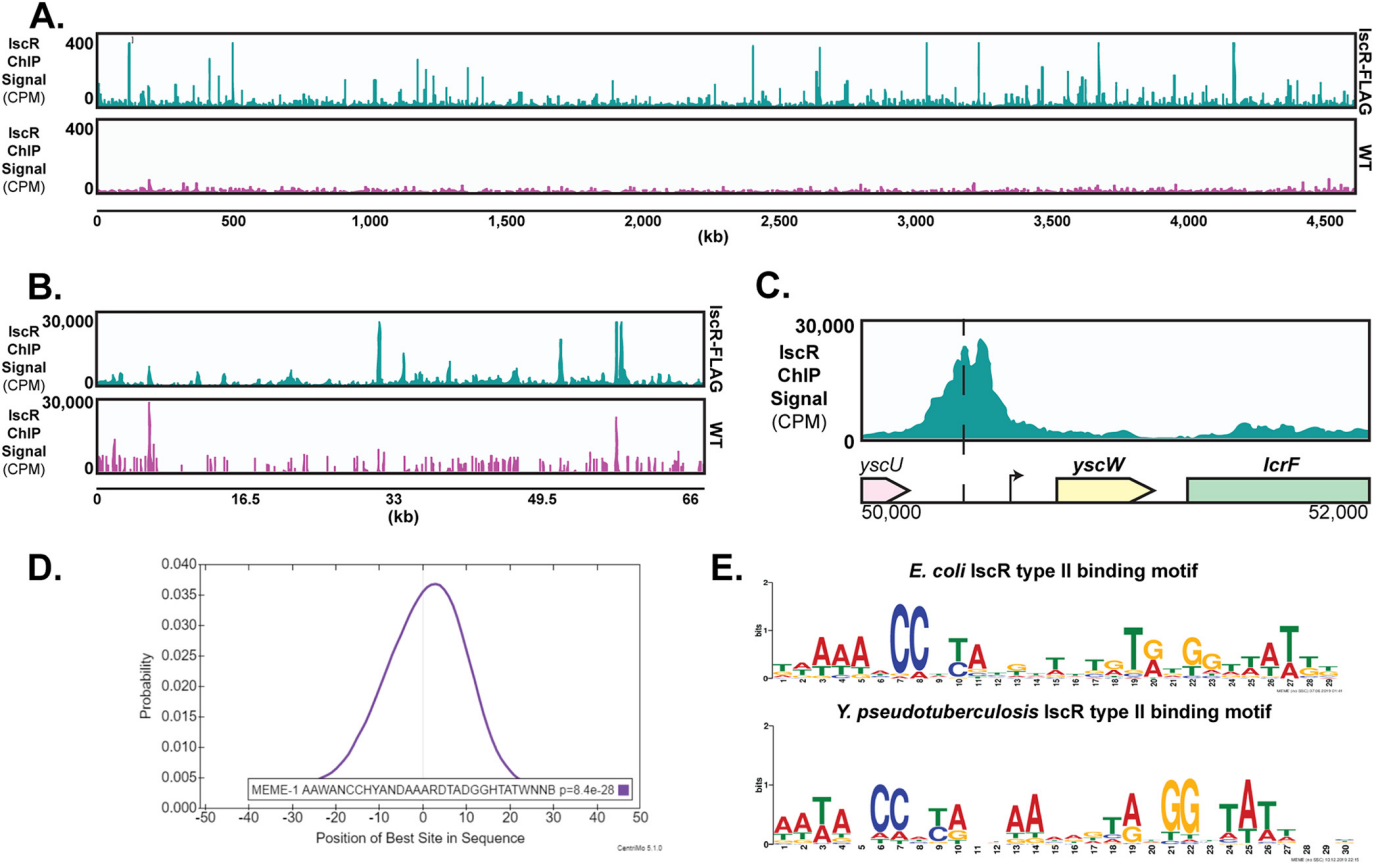
ChIP-Seq reveals IscR to be a global regulator in *Yersinia.* (A) ChIP-Seq analysis reveals enrichment of IscR binding sites throughout the genome following anti-FLAG pulldown. The first track (blue) represents read coverage of IscR-bound sequences throughout the Y. pseudotuberculosis IscR-3-xFLAG chromosome from three biological replicates, shown as count per million reads (CPM). The second track (pink) represents sequencing reads from WT Y. pseudotuberculosis (negative control). (B) ChIP-Seq plots illustrating read coverage of the pYV virulence plasmid (not scaled to the size of the genome). The *y* axis for the pYV plasmid is significantly higher than for the chromosome because pYV copy number is expected to be high under these conditions (96). (C) ChIP-Seq sequence read peaks mapped to the *yscW-lcrF* promoter. The *x* axis indicates the genomic position of the ChIP-Seq peaks. Dashed lines correspond to the center of the known 30-bp IscR type II binding site. The arrow indicates the transcriptional start site (39). (D) Motif that is overrepresented within 100 nucleotides of the zenith of IscR ChIP-Seq sequence read peaks. This motif was identified in 175 peaks out of 176 peaks. The motif representing the type II motif characterized in E. coli K-12 MG1655 is shown as reference. (E) The motif illustrated in Fig. 5D has a high probability of being located at or near the peak summit (Centri-Mo).

In order to identify the genes directly regulated by IscR, we carried out *in vivo* genome-wide detection of IscR binding sites via ChIP-Seq, using an anti-FLAG antibody and a Y. pseudotuberculosis strain expressing FLAG-tagged IscR (see Fig. S1 in the supplemental material; see Materials and Methods). Wild-type Y. pseudotuberculosis was used as a negative control since the FLAG antibody should not pull down IscR-DNA complexes in the absence of the affinity tag. A total of 295 unique regions of the genome were enriched during the FLAG pulldown (Fig. 5A and B). Of these ChIP-Seq peaks, 176 fell within 500 nucleotides upstream of an open reading frame (ORF) start codon and no more than 100 nucleotides downstream of a start codon, potentially within a regulatory region controlling transcription. Out of these 176 peaks, 173 are found on the chromosome, and three on the pYV virulence plasmid. A total of 37 peaks fell in between divergent genes, and such ChIP-Seq peaks were assigned to both genes (encoded on the sense and antisense strands) for our preliminary analysis. Therefore, the 176 identified peaks fell within the predicted regulatory regions of 213 transcription units (TUs) encoding 401 individual genes (Data Set S2). Of these 213 TUs, 46 contain genes found in cluster II (upregulated by iron starvation) and 40 contain genes found in cluster VI and VII (downregulated by iron starvation). The remaining 127 transcription units associated with a ChIP-Seq peak were not found in our cluster analysis, as they were not differentially expressed in response to iron. These data show that 40% of the IscR binding sites identified by our ChIP-Seq analysis were upstream of genes responsive to iron under aerobic conditions.

10.1128/mBio.00633-21.1 FIG S1 3xFLAG-tagged IscR rescues an *iscR* deletion. (A) Whole-cell extracts from WT Y. pseudotuberculosis or a strain harboring a chromosomally encoded 3xFLAG-tagged IscR were visualized using anti-FLAG or anti-RpoA antibodies. (B) To measure the relative efficiency of the Ysc T3SS, *Yersinia* strains were grown under T3SS-inducing conditions, and secreted proteins precipitated by trichloroacetic acid were visualized using Coomassie blue. Relative amounts of the T3SS effector protein YopE were quantified by densitometry compared to a spiked-in BSA protein control. The averages of three biological replicates ± standard deviations are shown. (C) *Yersinia* strains were grown under T3SS-inducing conditions, and relative *iscS* mRNA levels were evaluated by qPCR and normalized to 16s rRNA. The average of three biological replicates ± standard deviation is shown. ***, *P* < 0.001; **, *P* < 0.01; n.s., not significant (one-way ANOVA with Dunnett’s *post hoc* test). Download FIG S1, PDF file, 0.2 MB. Copyright © 2021 Balderas et al. 2021 Balderas et al. https://creativecommons.org/licenses/by/4.0/ This content is distributed under the terms of the Creative Commons Attribution 4.0 International license.

10.1128/mBio.00633-21.9 DATA SET S2 ChIP-Seq data assessing genome wide enrichment of IscR binding sites in Y. pseudotuberculosis. Download Data Set S2, XLSX file, 0.1 MB. Copyright © 2021 Balderas et al. 2021 Balderas et al. https://creativecommons.org/licenses/by/4.0/ This content is distributed under the terms of the Creative Commons Attribution 4.0 International license.

The most well-documented IscR binding site in *Yersinia* is the type II site found 367 nucleotides upstream of the *yscW* start codon (19). A ChIP-Seq peak was detected in this region, with the pinnacle of the peak centering 364 nucleotides upstream of *yscW-lcrF* (Fig. 5C). We used MEME suite tools to probe for overrepresented sequences near the center of ChIP-Seq peaks (41). MEME analysis revealed identifiable IscR type II binding sequences in 175 out of the 176 ChIP-Seq peaks positioned within a putative regulatory region, with the predicted binding sequences highly correlated with the center of the ChIP-Seq peak (CentriMo *P* value, 8.4E−1028; Fig. 5D). The overrepresented sequence from these IscR-enriched sites strongly resembles the consensus IscR type II site from E. coli (Fig. 5E) (28). No significant ChIP-Seq peaks were detected at predicted IscR type I sites in *Yersinia* (Fig. S2A and B). This may be because holo-IscR does not cross-link as well or bind with high enough affinity as apo-IscR, and the resulting peaks are difficult to detect over the signals of apo-IscR binding. Taken together, these data indicate that our 3xFLAG-IscR ChIP was able to enrich for IscR type II binding sites, but not type I binding sites, under the conditions used. Importantly, genes driven by type II motif-containing promoters are predicted to be regulated by IscR under iron-limited and aerobic conditions, such as those encountered by *Yersinia* during disseminated infection and therefore potential virulence factors.

10.1128/mBio.00633-21.2 FIG S2 Global IscR ChIP-seq analysis fails to identify predicted IscR type I sites. (A) Known E. coli IscR type I binding sites were used to generate an IscR type I binding motif using MEME suite tools. The Y. pseudotuberculosis IP2666 genome was scanned for IscR type I sites using FIMO specifically upstream of the *nfuA*, *iscRSUA*, *erpA*, and *DN756_20960_cysE* promoters. The predicted sequences were aligned to the E. coli consensus IscR type I binding motif. (B) IscR ChIP-seq plots illustrating read coverage of IscR binding peaks assigned to the promoter of *nfuA*, *iscRSUA*, *erpA*, and *DN756_20960*_*cysE* from all three replicates combined. Dashed lines correspond to the zenith of the predicted 29-bp IscR type I motifs. Download FIG S2, PDF file, 0.1 MB. Copyright © 2021 Balderas et al. 2021 Balderas et al. https://creativecommons.org/licenses/by/4.0/ This content is distributed under the terms of the Creative Commons Attribution 4.0 International license.

**FIG 6:**
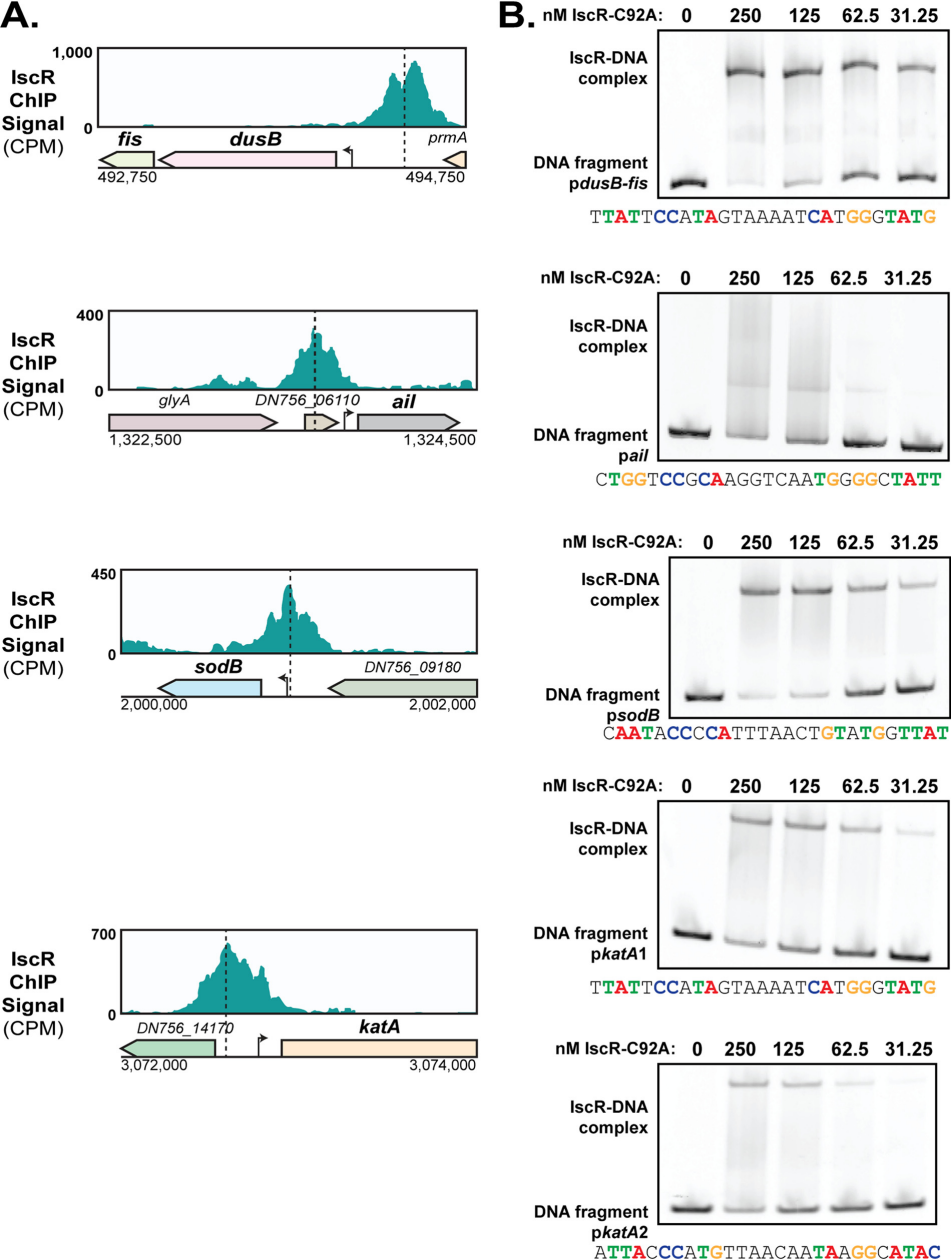
IscR binds to the promoters of *dusB-fis*, *ail*, *sodB*, and *katA in vivo* and *in vitro.* (A) IscR-enriched sequence reads located within the promoter regions of *dusB-fis*, *ail*, *sodB*, and *katA*. The *y* axis indicates read count, while the *x* axis indicates the genomic position of the ChIP-Seq peaks. Dashed lines correspond to the identified 30-bp IscR type II motif sequence. The arrows indicate previously identified transcriptional start sites (39). (B) Purified E. coli IscR-C92A was used for electrophoretic mobility shift assays (EMSAs) using DNA from the promoter regions of *dusB-fis*, *ail*, *sodB*, and *katA*. The predicted IscR type II binding site is noted below each EMSA, and critical nucleotides are highlighted in color. Note the *katA* promoter template was split into two distinct fragments.

In order to validate our *in vivo* IscR binding results, we carried out *in vitro* binding studies on the identified IscR binding sites upstream of the *katA* and *sodB* antioxidants as well as the known virulence genes *fis* and *ail* (Fig. 6A). IscR has been shown to regulate antioxidant genes in other bacterial pathogens (23, 42, 43). The gene *fis*, which is part of the *dusB-fis* operon, encodes a nucleoid-associated protein involved in resistance to oxidative stress (44). The protein Ail is important for tight attachment to host cells, type III secretion, and resistance against serum killing (45, 46). Although, unlike the *katA* and *sodB* genes, expression of *fis* and *ail* was not responsive to iron, we included them in our analysis because of their known roles in extraintestinal infection (44, 46). Since the promoters of these IscR targets are predicted to encode an IscR type II site, we used purified IscR-C92A (apo-locked IscR) to assess binding *in vitro*. *In vitro* electrophoretic mobility shift assays (EMSAs) showed that apo-IscR binds to these promoters, although the binding to the *ail* promoter appeared weaker (Fig. 6B). Indeed, the binding site predicted upstream of *ail* is missing key residues known to be important for IscR binding. A very large band shift was observed when assessing IscR binding to the *katA* promoter fragment (unpublished observations), indicating the possibility of more than one IscR binding site in this region. Indeed, when the *katA* promoter fragment was split into two, each with a bioinformatically identifiable IscR type II motif, binding to both fragments was observed. Collectively, these data demonstrate that IscR binds both *in vitro* and *in vivo* to the promoters of genes known or predicted to be involved in virulence.

### IscR binding sites are conserved in human-pathogenic *Yersinia*.

**FIG 7:**
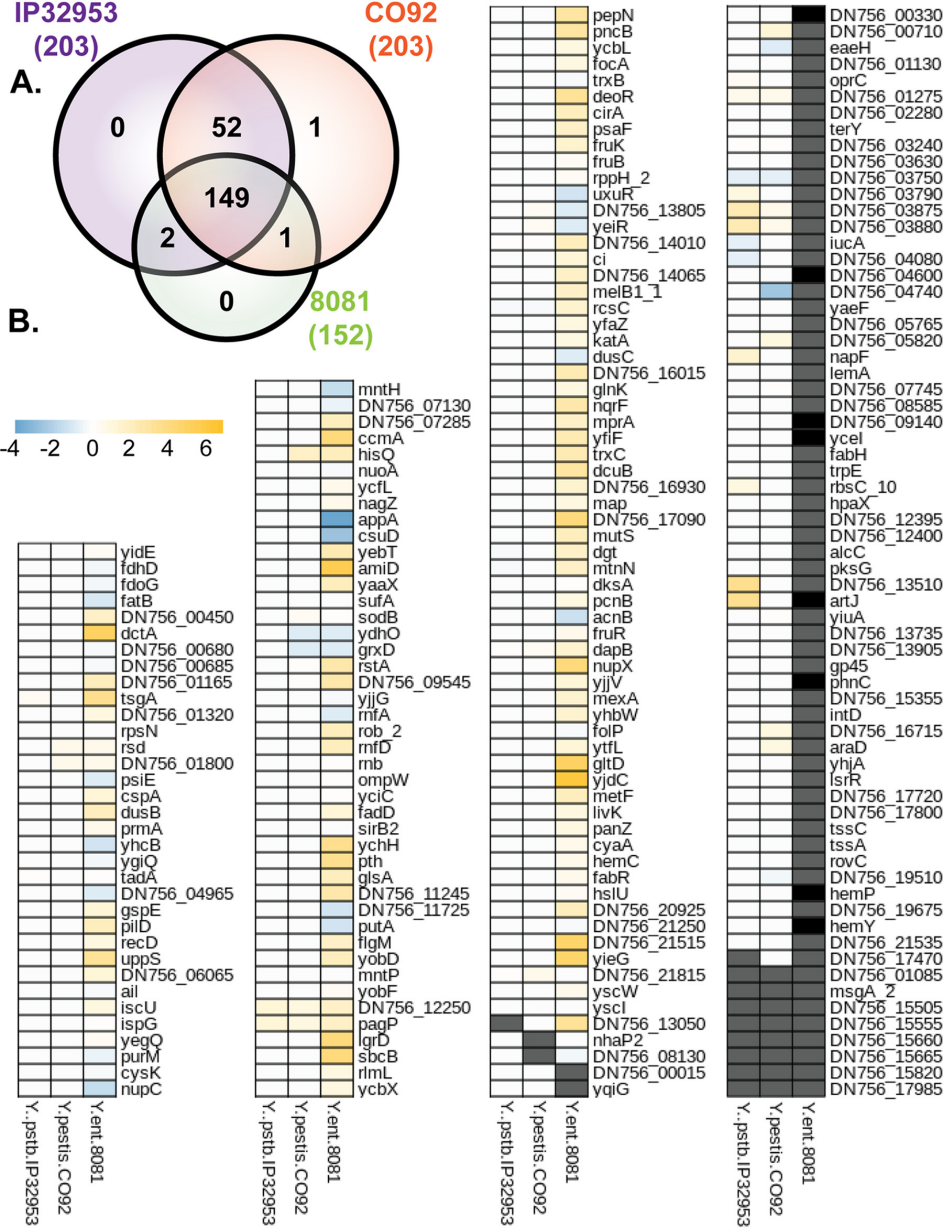
IscR binding site sequences are highly conserved in human-pathogenic *Yersinia.* (A) The first gene of the 213 transcription units identified in this study as being targets of IscR in Y. pseudotuberculosis IP2666 were used to identify orthologs in Y. pseudotuberculosis IP32953, Yersinia pestis CO92, and Yersinia enterocolitica 8081. (B) Heatmap showing similarity of IscR binding sites in Y. pseudotuberculosis IP32953, Y. pestis CO92, and Y. enterocolitica 8081 compared to Y. pseudotuberculosis IP2666, in the promoters of the 213 IscR transcription units. Blue boxes indicate a bioinformatically identified IscR type II binding motif predicted to enable stronger binding in the IP32953, CO92, or 8081 strain compared to the IP2666 strain. Yellow boxes indicate a motif predicted to bind IscR more weakly in strain IP32953, CO92, or 8081 compared to IP2666. White boxes indicate the predicted IscR binding site is similar in IP32953, CO92, or 8081 compared to IP2666. Gray boxes indicate no ortholog was found in the strain. Black boxes indicate an ortholog was identified but no IscR binding site was predicted by MEME suite tools. Values represent absolute log difference between MEME-FIMO-pvalue of known IscR binding site to orthologous IscR binding site.

We previously showed that IscR regulated the T3SS in both Y. pestis and Y. pseudotuberculosis, suggesting conservation of IscR regulation between these two species (9). To assess the degree of conservation of the identified IscR binding sites in the human-pathogenic *Yersinia* species, we carried out a comparative genomics analysis. We first searched for orthologs of the first gene of each of the 213 TUs containing an upstream IscR *in vivo* binding site in Y. pseudotuberculosis IP2666 (Data Set S3). Out of these 213 TUs, we could identify 203 orthologs of the first gene in each TU in Y. pseudotuberculosis IP32953, 203 in Y. pestis CO92, and 152 in Y. enterocolitica 8081 (Fig. 7A), which is more distantly related to Y. pseudotuberculosis compared to Y. pestis. In order to assess conservation of IscR binding sites among these four strains, MEME suite was utilized to assess the similarity of identified IscR binding motifs to the IscR type II consensus motif generated from this study (Fig. 5E). Of the TUs for which an ortholog could be identified in at least one of the other *Yersinia* strains, the majority contained an upstream identifiable IscR type II binding motif. If orthologous type II motif sequences in two different *Yersinia* strains are functionally conserved in terms of IscR regulatory control, then the distance between the motif and the downstream gene should be similar between the two species. Indeed, the predicted IscR binding site for orthologous genes were of similar distance from the downstream start codon, with more similarity among the Y. pseudotuberculosis and Y. pestis strains than between Y. pseudotuberculosis and Y. enterocolitica (Fig. S3A). In addition, the IscR binding sites overall are highly conserved among strains IP2666, IP32953, and CO92, although many diverge in strain 8081 (Fig. S3B). In order to visualize the conservation of IscR type II motifs in *Yersinia*, a heatmap was generated illustrating the retainment of critical nucleotides that have been shown to be important for IscR binding (Fig. 7B) (28). Importantly, there was very high conservation of IscR type II motifs between Y. pseudotuberculosis and Y. pestis. Out of the 203 transcription units whose putative regulatory regions were bound by IscR in Y. pseudotuberculosis IP2666 and conserved in Y. pestis CO92, 173 (∼85%) had IscR type II motif sequences in their promoters that were 100% conserved between strains IP2666 and CO92, while 17 (∼8%) contained only differences in nucleotides not critical for IscR binding. Interestingly, the IscR binding sites that were among the most conserved between all three *Yersinia* species included those within the promoters of *sodB*, *katA*, the *suf* operon, *pagP*, *ompW*, *yscW-lcrF*, *dusB-fis*, and *ail*.

10.1128/mBio.00633-21.3 FIG S3 Conservation of IscR binding sites in Y. pseudotuberculosis (IP2666, IP32953), Y. pestis (CO92), and Y. enterocolitica (8081). (A) Distance of the identified IscR binding site in Y. pseudotuberculosis (IP2666) from the start codon of each transcription unit member of the IscR regulon is plotted versus the distance of the predicted IscR binding site from the start codon of the identified ortholog in Y. pseudotuberculosis (IP32953), Y. pestis (CO92), or Y. enterocolitica (8081). (B) The log_10_ FIMO *P* value of the identified IscR binding site in Y. pseudotuberculosis (IP2666) is plotted versus the log_10_ FIMO *P* value of the predicted IscR binding site upstream of identified orthologs in Y. pseudotuberculosis (IP32953), Y. pestis (CO92), or Y. enterocolitica (8081). Download FIG S3, PDF file, 0.3 MB. Copyright © 2021 Balderas et al. 2021 Balderas et al. https://creativecommons.org/licenses/by/4.0/ This content is distributed under the terms of the Creative Commons Attribution 4.0 International license.

10.1128/mBio.00633-21.10 DATA SET S3 Comparative genomics of IscR binding sites in Y. pseudotuberculosis IP2666, Y. pseudotuberculosis IP32953, Y. pestis CO92, and Y. enterocolitica 8081. Download Data Set S3, XLSX file, 0.08 MB. Copyright © 2021 Balderas et al. 2021 Balderas et al. https://creativecommons.org/licenses/by/4.0/ This content is distributed under the terms of the Creative Commons Attribution 4.0 International license.

### Integration of transcriptome profiling with identified IscR binding sites.

To determine which genes are directly regulated by IscR in Y. pseudotuberculosis, we collated the ChIP-Seq results with our published RNA-Seq data carried out under the same aerobic, non-iron-starved conditions used for the IscR chromatin precipitation (19) (Data Set S2). Under this condition, only 18 out of the 213 putative IscR regulatory sites found to be associated with an IscR binding site drove IscR-dependent changes in expression of predicted transcription units. Three of these 18 TUs, DN756_21815-DN756_21820, *yscW-lcrF*, and *yscIJKL*, are carried on the pYV virulence plasmid and were downregulated in the *iscR* mutant. We have extensively validated direct regulation of the *yscW*-*lcrF* operon by IscR (9, 19) (Fig. 5C). The *yscIJKL* T3SS genes are carried within the *virC* operon, *yscABCDEFGHIJKL* (47), which is controlled by the T3SS master regulator LcrF (48). Since *lcrF* transcription is directly regulated by IscR (19), further experiments are needed to determine whether alternative transcriptional start sites that are regulated by LcrF or IscR exist for the *virC* operon. DN756_21815 and DN756_21820 are a hypothetical gene and pseudogene, respectively, and are carried on the pYV virulence plasmid. Interestingly, deletion of DN756_21815 and DN756_21820 had a small but significant effect on secretion of the T3SS effector protein YopE into culture supernatant (Fig. S4). The remaining 15 TUs are encoded on the chromosome and include *sodB*, *ail*, and *fis*. A number of ChIP-Seq studies have shown that transcription factors exhibit binding to promoters whose genes are differentially regulated by the transcription factor under some but not all conditions tested, including the condition used for ChIP-Seq (49, 50). Therefore, it was not surprising to see limited overlap between the ChIP-Seq data and RNA-Seq from only one culture condition.

10.1128/mBio.00633-21.4 FIG S4 Deletion of DN756_21815 and DN756_21820, identified gene targets of IscR, results in a small but significant decrease in type III secretion. (A) Read coverage of IscR-binding peaks proximal to *DN756_21815_DN756_21820*. Counts per million reads (CPM) are plotted versus the genomic position. Dashed lines correspond to the predicted 30-bp IscR type II motif. Arrows indicate transcriptional start sites (48). (B) Expression of *DN756_21815*, and *DN756_21820* genes under various iron conditions as measured by RNA-seq. Reads are represented by trimmed mean of M-values (TMM) of WT (black) and Δ*iscR* strains (gray) grown in M9 minimal medium containing FeSO_4_ (non-iron-starved [NIS]), iron starved in Chelex-treated M9 minimal medium with no iron source added back (Chelex) or supplemented with 5 mM hemin (+Heme) or FeSO_4_ (+FeSO_4_). **, *P* < 0.01; ***, *P* < 0.001; ****, *P* < 0.0001 (EdgeR with a corrected FDR *post hoc* test). (C) *Yersinia* strains were grown under rich media, T3SS-inducing conditions. The secretome of these cultures was visualized with Coomassie blue. The effector protein, YopE, was quantified by densitometry relative to the WT control to measure the relative efficiency of the Ysc T3SS. The average of five biological replicates ± standard deviation is shown. ****, *P* < 0.0001; *, *P* < 0.05 (one-way ANOVA with Dunnett’s *post hoc* test). Download FIG S4, PDF file, 0.3 MB. Copyright © 2021 Balderas et al. 2021 Balderas et al. https://creativecommons.org/licenses/by/4.0/ This content is distributed under the terms of the Creative Commons Attribution 4.0 International license.

We assessed whether our transcriptome analysis of iron-starved *Yersinia* might reveal additional genes associated with *in vivo* IscR binding whose expression is regulated by IscR. Indeed, 86 TUs with an upstream IscR binding site were found to contain genes differentially expressed in the presence and absence of IscR under at least one of the RNA-Seq conditions (Data Set S2). We carried out a cluster of orthologous genes (COG) analysis on genes of the IscR direct regulon whose promoters contain an IscR enrichment site (Fig. S5A), functional regulon genes associated with a ChIP-Seq peak that showed differential expression by IscR in at least one condition tested (Fig. S5B), and the indirect regulon whose expression depends on IscR but are not associated with a ChIP-Seq peak (Fig. S5C). The direct regulon was enriched for coenzyme and lipid metabolism as well as inorganic iron uptake compared to the indirect regulon, while the indirect regulon was enriched for biosynthesis (amino acid, nucleotide, and carbohydrate metabolism), energy production, and the T3SS (virulence plasmid). Included in the functional regulon were genes whose expression patterns are consistent with apo-IscR induction or repression in our RNA-Seq analysis and are therefore predicted to be directly controlled by IscR during disseminated infection: the *suf* operon; the metal transporters *iucABCDiutA*, *alcC*, *cirA*, *fhuC*_1, *oprC*, and *yiuA*; the cell envelope enzymes *pagP*, *amiD*, and *ydhO*; and the oxidative stress associated *sodB*, *katA*, and *ompW*. However, many genes within the functional regulon did not display an expression pattern consistent with apo-IscR activation or repression yet showed differential expression in response to IscR under at least one RNA-Seq condition. This is consistent with other reports that identified ChIP-Seq peaks for genes differentially regulated by their respective transcription factors only under certain environmental conditions (49, 50).

10.1128/mBio.00633-21.5 FIG S5 COG analysis of the IscR regulon, functional regulon, and indirect regulon. Clusters of Orthologous Groups of proteins (COG) analysis of genes with an IscR ChIP-Seq peak (A), genes with an IscR ChIP-Seq peak and are differentially expressed between WT and the *iscR* mutant under at least one tested condition (B), and genes differentially expressed between WT and the iscR mutant but have no IscR ChIP-Seq peak (C). Download FIG S5, PDF file, 0.3 MB. Copyright © 2021 Balderas et al. 2021 Balderas et al. https://creativecommons.org/licenses/by/4.0/ This content is distributed under the terms of the Creative Commons Attribution 4.0 International license.

### IscR directly regulates the *suf* operon, which contributes to type III secretion under iron-limiting conditions.

**FIG 8:**
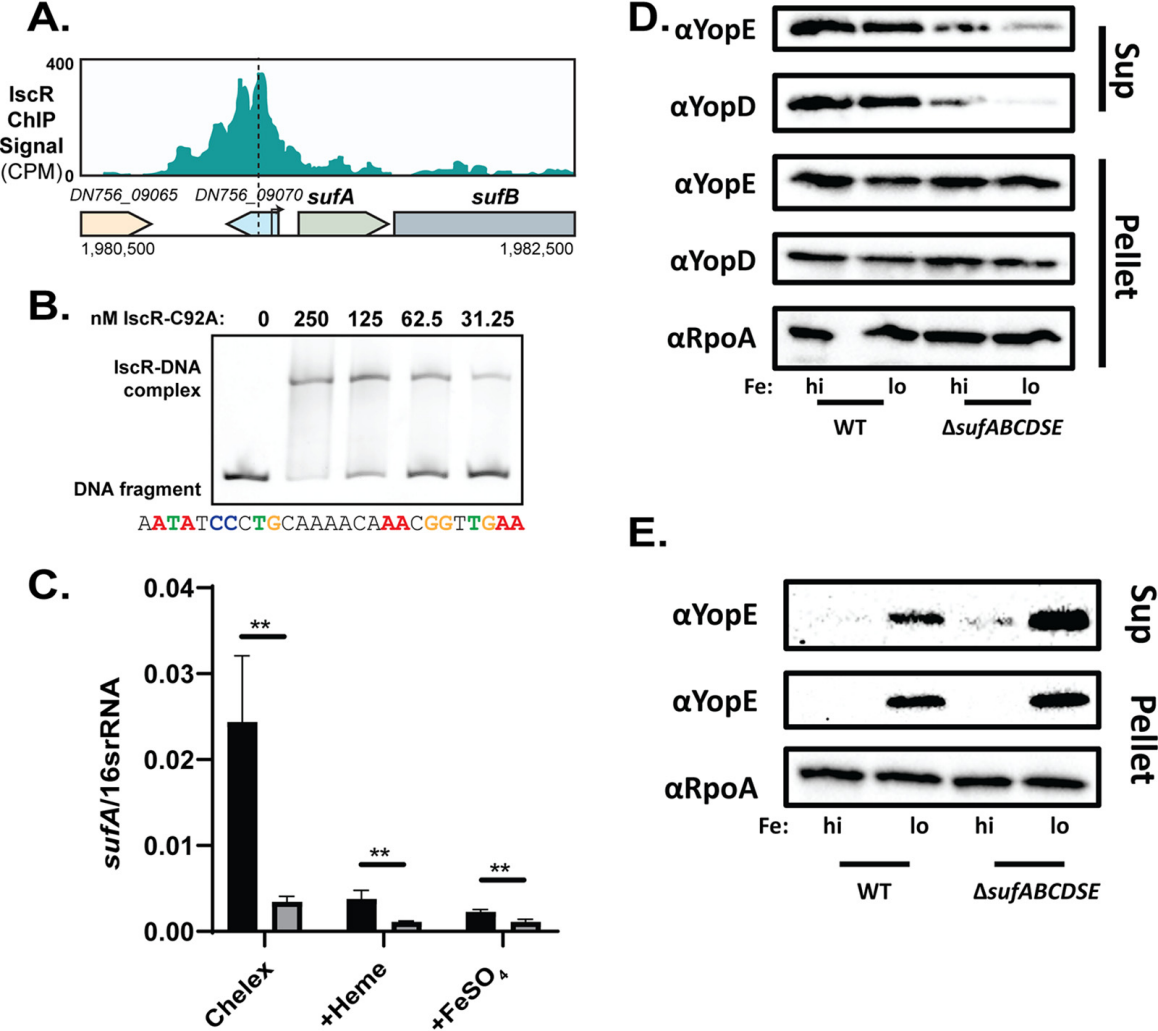
IscR directly regulates the Suf Fe-S cluster biogenesis pathway, which is important for type III secretion activity specifically under aerobic conditions. (A) IscR-enriched sequence reads located within the promoter regions of *sufABCDSE*. The *y* axis indicates read count, while the *x* axis indicates the genomic position of the ChIP-Seq peaks. Dashed lines correspond to the identified 30-bp IscR type II motif sequence. Arrows indicate previously identified transcriptional start site (39). (B) Purified E. coli IscR-C92A was used for electrophoretic mobility shift assays (EMSAs) using DNA from the promoter regions of *sufABCDSE*. The predicted IscR type II binding site is noted below the EMSA, and critical nucleotides are highlighted in color. (C) Expression of *sufA* relative to 16s rRNA as measured by qRT-PCR of WT (black) and Δ*iscR* strains (gray). **, *P* < 0.01 (one-way ANOVA with Dunnett’s *post hoc* test). Average of three independent experiments ± standard deviation is shown. (D and E) WT and Δ*sufABCDSE* cultures were iron starved in Chelex-treated M9 minimal medium and supplemented with either 3.6 μM FeSO4 (high [hi]) or 0.036 μM FeSO4 (low [lo]) before type III secretion was induced under aerobic (D) or anaerobic (E) conditions. Proteins secreted into culture supernatant (Sup) and precipitated with trichloroacetic acid were probed with antibodies for YopE and YopD T3SS cargo proteins. Cell lysates were probed with antibodies for RpoA, YopE, and YopD. One representative experiment out of three biological replicates is shown.

The validated IscR binding site in the *suf* promoter exhibited 100% conservation among all human-pathogenic *Yersinia* (Fig. 7B), yet the role of the *suf* operon has never been studied in *Yersinia*. We observed a ChIP-Seq peak in the *suf* promoter with an identifiable IscR type II binding motif (Fig. 8A), as well as *in vitro* binding of IscR to the *suf* promoter using an EMSA (Fig. 8B). Furthermore, the *suf* operon was differentially expressed by IscR following iron starvation (Fig. S6), when IscR levels are high and Fur repression is predicted to decrease (51, 52). Indeed, *sufABCDSE* were found to be expressed 3- to 11-fold more in wild-type Y. pseudotuberculosis than in the Δ*iscR* mutant during iron starvation (Fig. 8C and Fig. S6). Together, these data strongly suggest that apo-IscR binds to a type II binding motif sequence in the *suf* promoter, activating *suf* operon expression.

10.1128/mBio.00633-21.6 FIG S6 Expression of the *suf* operon is modulated by iron and requires IscR. Expression of *sufA*, *sufB*, *sufC*, *sufD*, *sufS*, and *sufE* genes under various iron conditions as measured by RNA-seq. Reads are represented by trimmed mean of M-values (TMM) of WT (black) and Δ*iscR* strains (gray) grown in M9 minimal medium containing 3.6 μM FeSO_4_ (non-iron-starved [NIS]), iron starved in Chelex-treated M9 minimal medium with no iron source added back (Chelex) or supplemented with 5 mM hemin (+Heme) or 3.6 μM FeSO_4_ (+FeSO_4_).**, *P* < 0.01; ***, *P* < 0.001; ****, *P* < 0.0001 (EdgeR with a corrected FDR *post hoc* test). Download FIG S6, PDF file, 0.1 MB. Copyright © 2021 Balderas et al. 2021 Balderas et al. https://creativecommons.org/licenses/by/4.0/ This content is distributed under the terms of the Creative Commons Attribution 4.0 International license.

We constructed a Δ*sufABCDSE* Y. pseudotuberculosis mutant, which exhibited normal growth and motility (Fig. S7A and B). Surprisingly, this mutant had a significant type III secretion defect under aerobic conditions, when the Suf system is the dominant Fe-S cluster biogenesis pathway (Fig. 8D and E). These data suggest that an Fe-S cluster-containing protein is important for T3SS expression or activity. As the *suf* mutant did not exhibit a defect in T3SS gene expression (Fig. S7C), these data indicate that the Suf pathway contributes to type III secretion in a posttranslational manner. The Isc pathway is the dominant Fe-S cluster biogenesis pathway under anaerobic conditions. However, we were unable to test whether the Isc pathway was required for type III secretion under anaerobic conditions because we were unsuccessful in deleting *iscSUA*, suggesting these are essential genes. Together, these data suggest that IscR regulates the *Yersinia* T3SS in two distinct ways: through direct control of the LcrF T3SS master regulator and through direct control of the Suf pathway that matures an Fe-S cluster protein critical for T3SS activity.

10.1128/mBio.00633-21.7 FIG S7 The Suf pathway does not affect motility or growth. (A) Iron-starved Y. pseudotuberculosis were either kept in iron-starved Chelex-treated M9 minimal medium with no iron source added back (Chelex) or supplemented with 3.6 μM FeSO_4_ (+FeSO_4_). Cultures were grown at 26°C for 10 h, and optical density at 600 nm was measured every hour. Non-iron-starved (NIS) samples were never exposed to Chelex-treated M9. (B) The indicated strains were spotted onto motility agar (1% tryptone, 0.25% agar) and were grown at 26°C. The diameters of the colonies were measured 24 h and 48 h later and used to calculate percent motility. Graphs represent three biological replicates. ****, *P* < 0.0001 (one-way ANOVA with Dunnett’s *post hoc* test). (C) Expression of *lcrF*, *iscR*, and *yopE*, relative to 16S rRNA, following iron-starved conditions in M9 minimal medium at 37°C, as measured by qPCR. Black bars represent cultures which were iron starved and supplemented with 3.6 μM FeSO_4_ before induction of the type III secretion system, while gray bars represent cultures that received 0.036 μM FeSO_4_ before induction of the type III secretion system. Average of three independent experiments ± standard deviation is shown. ***, *P* < 0.001; **, *P* < 0.01; *, *P* < 0.05 (one-way ANOVA with Dunnett’s *post hoc* test). Download FIG S7, PDF file, 0.2 MB. Copyright © 2021 Balderas et al. 2021 Balderas et al. https://creativecommons.org/licenses/by/4.0/ This content is distributed under the terms of the Creative Commons Attribution 4.0 International license.

## DISCUSSION

**FIG 9:**
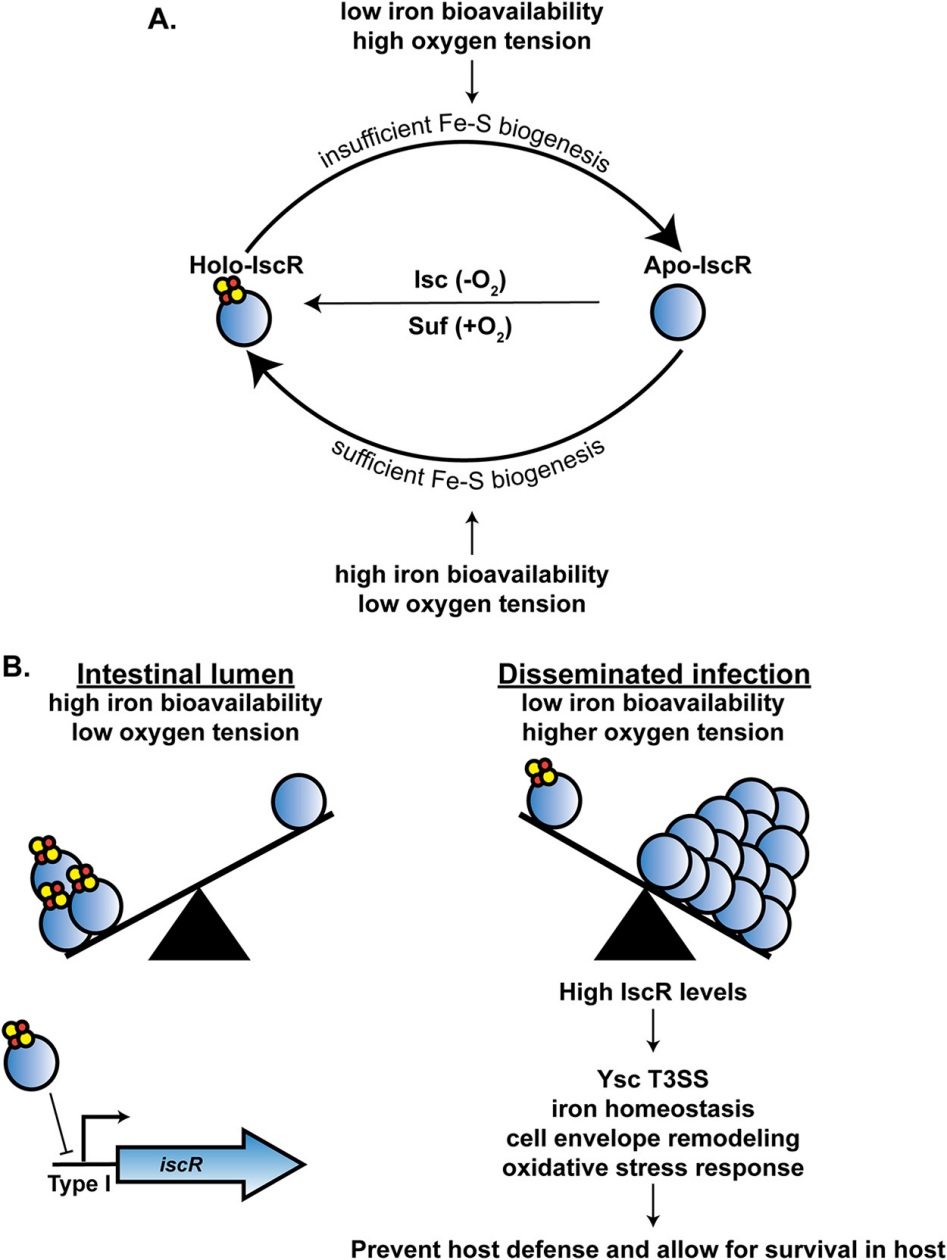
Model of how iron modulates IscR levels and leads to differential expression of IscR-dependent virulence factors. (A) The Suf system loads Fe-S clusters onto IscR under aerobic conditions, while the Isc system is more active under anaerobic conditions. Iron bioavailability and oxygen tension affect the holo-/apo-IscR ratio. (B) Low iron and oxidative stress, which are encountered by *Yersinia* during disseminated infection, lead to increased Fe-S cluster demand. This leads to a decrease in holo-IscR, subsequent derepression of overall IscR expression, and an increase in apo-IscR levels. Apo-IscR directly regulates genes involved in the T3SS, oxidative stress resistance, cell envelope remodeling, iron homeostasis, and other processes, enabling virulence.

IscR and iron are both critical factors in the pathogenesis of *Yersinia* and a number of other bacteria (9, 14, 20, 21, 53). In this study, we present the first characterization of the IscR direct regulon. We used ChIP-Seq and RNA-Seq analysis to identify *Yersinia* genes directly regulated by IscR in response to changes in iron availability. Our data suggest that IscR allows *Yersinia* to couple sensing of iron starvation, a condition experienced by the bacterium during extraintestinal infection, to control of genes involved in iron homeostasis, cell envelope modification, oxidative stress response, as well as T3SS expression and activity (Fig. 9). Over 93% of *in vivo* IscR binding sites identified in Y. pseudotuberculosis were conserved in Y. pestis, suggesting that Y. pestis retained IscR as an important regulator after its evolution from foodborne pathogen to plague agent.

In this study, we show that complete iron starvation induces IscR mRNA and protein levels in *Yersinia* under aerobic conditions. Previously, iron limitation was shown to induce IscR levels in both E. coli and Vibrio vulnificus (33, 54). Iron starvation not only limits the amount of free iron that can incorporate into Fe-S cofactors but can also affect the function of the Fe-S cluster biogenesis machinery (55). Ultimately, iron starvation leads to a reduction of Fe-S cluster availability causing a shift to more apo-IscR compared to holo-IscR (Fig. 9). Since holo-IscR represses transcription of the *isc* operon, this shift leads to derepression of the *isc* operon under iron-starved conditions (31). We previously found that supplementing *Yersinia* with as little as 0.036 μM FeSO_4_ prevented this derepression of *iscR*, suggesting why in our earlier study IscR levels were not iron responsive under aerobic conditions (9). However, we show here that following prolonged iron starvation, IscR and IscR target genes are indeed responsive to either inorganic or heme iron. These data suggest that during infection, when the availability of free iron is vanishingly low and is further reduced by inflammatory pathways (39, 40), IscR levels should fluctuate in response to the ability of *Yersinia* to scavenge iron.

Two important categories of genes found to be regulated by iron and IscR were involved in cell envelope modification and the oxidative stress response. *Yersinia* modifies its LPS during growth at 37°C such that its LPS is less stimulatory to the endotoxin receptor Toll-like receptor 4 (TLR4) (56–59). Interestingly, we found in this study that a number of *Yersinia* LPS biosynthesis and modification enzymes genes are upregulated upon iron starvation. Of these genes, only *pagP*, which encodes an outer membrane lipid A palmitoyltransferase, was found to be directly regulated by IscR. The *pagP* promoter contained an IscR type II motif that bound to IscR *in vivo*, and *pagP* expression was consistent with induction by apo-IscR. Salmonella PagP, which is regulated by magnesium through the PhoP/Q regulatory system (60), enables production of LPS lipid A that is less stimulatory to TLR4 (61–63). In contrast, Y. pestis has an inactive *pagP* allele, but when the Y. pseudotuberculosis functional *pagP* gene is inserted into Y. pestis, the LPS that is produced is more immunostimulatory (64). Therefore, how IscR affects LPS stimulation of TLR4 and other LPS sensors remains to be determined. To our knowledge, iron availability has not been previously shown to affect *Yersinia* LPS gene expression nor has IscR been shown to regulate LPS modifying genes in other bacterial species. In contrast, IscR has been implicated in controlling genes involved in the response to free radical stress in Vibrio vulnificus, Pseudomonas aeruginosa, and Burkholderia mallei (21, 23, 24, 43, 54). The *Yersinia* T3SS, which is positively regulated by IscR, inhibits phagocytic cell oxidative burst. Therefore, IscR induction of the T3SS should lead to a decrease in the amount of reactive oxygen species (ROS) encountered by *Yersinia* during infection. In this study, we also found that IscR directly repressed the genes encoding catalase, *katA*, superoxide dismutase, *sodB*, and the oxidative stress-associated OmpW (65). It is possible that IscR upregulation of the T3SS may be coupled to lower expression of KatA and SodB, since the bacteria would be less likely to encounter ROS stress when the T3SS is active.

We showed that IscR binds to the *sufABCDSE* promoter both *in vitro* and *in vivo* and that *suf* expression is consistent with apo-IscR induction of the *suf* promoter. This is in line with a previous study showing that IscR activates the E. coli *suf* operon upon exposure to the iron chelator dipyridyl (33). However, surprisingly, our data also showed that deletion of the *suf* operon leads to reduced Ysc T3SS activity. Loss of the *suf* operon did not affect T3SS gene expression or protein levels but instead affected secretion of T3SS cargo. This phenotype was observed under standard aerobic non-iron-starved conditions (data not shown) and was even more pronounced under iron-limited conditions where the *suf* operon becomes more important for synthesizing Fe-S clusters. Loss of the *suf* operon may affect the electron transport chain and lead to a proton motive force defect, which is required for T3SS activity (66). However, the *suf* mutant had no motility defect, which also requires the proton motive force. Under anaerobic iron-replete conditions, when the Isc pathway mediates Fe-S cluster biogenesis for the cell, the *suf* mutant displays normal type III secretion. Collectively, these results suggest that a *Yersinia* Fe-S cluster protein, matured by the Suf pathway under aerobic conditions, promotes T3SS activity. Interestingly, the Salmonella Isc pathway, but not the Suf pathway, was shown to be important for IscR-mediated repression of the SPI-1 T3SS and for virulence following oral infection. In contrast to the *Yersinia* T3SS, Salmonella expresses its SPI-1 T3SS in the intestine where it is required for entry into intestinal epithelial cells (20).

IscR positively regulates the T3SS master regulator LcrF, and type III secretion occurs only under anaerobic conditions in the absence, not the presence, of iron (9, 19). Therefore, it was surprising that only the *virC* structural operon (*yscCDEFGHKL*) but not other LcrF-regulated genes were significantly upregulated under our aerobic iron starvation conditions. Indeed, while mRNA levels of the *yscW-lcrF* operon trended up in the absence of iron, this difference was not statistically significant. In fact, some T3SS effector protein and chaperone genes (*yopO*, *yopK*, *yopH*, *yopJ*, and *sycDH*), the *yscM* T3SS regulatory gene, and the needle tip protein *lcrV* were expressed approximately two- to fourfold more after iron supplementation. However, importantly, most of these genes were downregulated ∼10- to 50-fold by deletion of *iscR*, demonstrating that the presence of an *iscR* gene had a much stronger effect on T3SS gene expression than changes in iron availability under aerobic conditions. Y. pseudotuberculosis transits from the small intestine to lymph tissue and vital organs during infection. In the intestines, *Yersinia* experiences an anaerobic iron-replete environment where it does not require its T3SS (67–69). In contrast, once *Yersinia* crosses the intestinal barrier and the T3SS becomes important for virulence, oxygen tension increases and host iron sequestration causes a drastically smaller amount of bioavailable iron. Collectively, our data show that IscR represses the *Yersinia* T3SS in the intestines and induces the T3SS once *Yersinia* disseminate.

Only about 40% of genes associated with an IscR binding site were found to be differentially regulated by IscR in at least one condition tested, and even fewer had expression patterns consistent with apo-IscR activation or repression. However, a recent study examining the regulons associated with five different two-component regulatory systems in E. coli noted the existence of genes whose promoters directly bound a transcription factor (TF) but showed differential expression by that TF only under certain conditions (50). The authors speculated that this is due to other regulators controlling such promoters and referred to these genes as exhibiting “hypothetical functional binding” by the TF, as other studies have also suggested (70). We postulate that *ail* and *dusB-fis* may fall into this category, as they are differentially expressed by IscR only under non-iron-starved conditions but show IscR binding to their promoters *in vitro* and *in vivo*. The *ail* gene encodes an adhesin that contributes to delivery of T3SS effector proteins into target host cells and mediates complement resistance (45, 46, 71, 72). The factor for inversion (Fis) nucleoid-associated protein influences the topological state of DNA and affects gene expression (73). Fis regulates virulence in a number of pathogens, including Y. pseudotuberculosis (44). In some organisms, Fis expression is regulated by growth phase, while in others it is regulated by specific environmental signals (73). For example, Salmonella Fis regulates the supercoiling of DNA encoding the SPI-1 and SPI-2 T3SSs in an opposite manner in response to oxygen (74, 75). In our study, we show that the *Yersinia fis* gene is expressed at higher levels under iron starvation conditions than after iron supplementation. In Y. pseudotuberculosis, Fis has been shown to be crucial for resistance to oxidative stress and colonization of the murine spleen and liver (44). Additional environmental signals, such as oxygen tension, should be examined for their ability to regulate *Yersinia ail* and *fis* to explore whether IscR regulation of these genes may be important under anaerobic conditions such as those found in the intestinal lumen.

The remaining 60% of genes associated with an IscR binding site were not found to be differentially expressed by IscR under any condition tested. Choudhary et al. categorized such genes as having “potentially nonfunctional binding” of the TF (50). The *in vivo* IscR binding associated with these genes either represents spurious, nonfunctional binding, or another regulator is preventing IscR from exerting an effect on gene expression under the conditions tested. Indeed, there is evidence that IscR regulates promoters also targeted by other TFs. For example, out of the 213 TUs targeted by IscR, 65 had a predicted Fur box in their corresponding regulatory region (unpublished observations). Additional RNA-Seq analysis of WT and Δ*iscR Yersinia* will help determine whether the “potentially nonfunctional binding” genes identified in this study are differentially expressed under certain conditions, for example anaerobic iron-replete conditions that mimic those encountered by *Yersinia* in the intestinal lumen where IscR is also necessary for colonization.

Fur in uropathogenic E. coli (UPEC) regulates genes found in pathogenicity islands and siderophores not present in commensal E. coli K-12 strain MG1655 (76). This difference in Fur regulon members from the two species could be due to the lack of the pathogenicity islands in commensal E. coli. Interestingly, UPEC Fur directly regulated genes that were also present in commensal E. coli, yet only UPEC Fur directly regulated this subset of genes. These data suggest that as species evolve and find new niches, regulons and transcription regulatory networks change to adapt to the environment. Current models suggest that Y. pestis evolved as recently as ∼1,500 years ago from Y. pseudotuberculosis through a series of DNA element acquisitions along with gene loss (77), making *Yersinia* an interesting model to study the IscR regulon. These genomic changes enabled a previously facultative foodborne pathogen to colonize and be transmitted through a flea vector. However, once inside the host, both Y. pseudotuberculosis and Y. pestis colonize lymph nodes and spread to deeper tissues (5, 36). In addition, while the two pathogens exhibit very different life cycles, there is evidence that, within lymph nodes, they both experience iron starvation (39, 40). For example, Y. pseudotuberculosis in the Peyer’s patches upregulate metal acquisition genes such as *hmuR*, *alcC*, *znuABC*, *mntH*, as well as others (39). These same genes are upregulated in our transcriptomic data upon iron starvation. The results of our comparative genomic analysis suggest that the IscR regulon is highly conserved in Y. pseudotuberculosis and Y. pestis. We speculate that IscR control of genes involved in iron homeostasis, virulence, stress response, and other pathways has provided a fitness benefit to Y. pestis during its evolution as the plague agent and was therefore retained. Furthermore, as a number of the IscR binding sites identified in Y. pseudotuberculosis are conserved in the Y. enterocolitica genome, we also speculate that IscR-regulated genes important for pathogenesis were present in the shared ancestor of Y. enterocolitica and Y. pseudotuberculosis/Y. pestis.

## MATERIALS AND METHODS

### Bacterial strains, plasmids, and growth conditions.

All strains used in this study are listed in Table 1. Y. pseudotuberculosis strains were grown in M9 minimal medium supplemented with Casamino Acids, referred to here as M9+3.6 μM FeSO_4_, at 26°C with shaking at 250 rpm, unless otherwise indicated (78). Non-iron-starved conditions were achieved by subculturing an M9+3.6 μM FeSO_4_ overnight culture to an optical density at 600 nm (OD_600_) of 0.2 in fresh M9+3.6 μM FeSO_4_, and shaking for 3 h at 37°C.

**TABLE 1:** Strains used in this study

Strain	Relevant genotype	Reference
IP2666 (WT)	Naturally lacks full-length YopT	97
IP2666 (Δ*iscR*)	*iscR* in-frame deletion of codons 2 to 156	19
IP2666 (IscR 3xFLAG)	In-frame C terminus 3xFLAG tag of chromosomal IscR	This work
IP2666 (Δ*sufABCDSE*)	*sufABCDSE* in-frame deletion retains first 10 codons of SufA and last 20 codons of SufE	This work
IP2666 (Δ*DN756_21815-* * DN756_21820*)	*DN756_21815-DN756_21820* in-frame deletion retains first 10 codons of DN756_21815 and last 10 codons of DN756_21820	This work
IP2666 (Apo-locked IscR)	IscR-C92A/C98A/C104A	19
IP2666 (Δ*flhD*)	WT strain with inactive *flhDC* from Yersinia pestis	19

Iron starvation was achieved by growing Y. pseudotuberculosis aerobically in M9 medium lacking iron treated with Chelex 100 resin to remove all traces of iron in acid-washed glassware, as previously described (9, 12). Specifically, iron-replete overnight cultures (M9+3.6 μM FeSO_4_) grown at 26°C aerobically were diluted to an OD_600_ of 0.1 into Chelex-treated M9 medium and grown for 8 h at 26°C aerobically with agitation. Cultures were then subcultured a second time to an OD_600_ of 0.1 in fresh Chelex-treated M9 and grown for 12 h at 26°C with agitation. Cultures were then subcultured to an OD_600_ of 0.1 into 20-ml Chelex-treated M9 medium with either no iron, 5 μM hemin, or 3.6 μM FeSO_4_ and grown for 3 h at 37°C with agitation.

### Construction of *Yersinia* mutant strains.

A 3xFLAG affinity tag was placed at the C terminus of IscR encoded at the native *iscR* chromosomal locus to facilitate detection of IscR with FLAG monoclonal antibody (see Fig. S1A in the supplemental material). The 3xFLAG affinity tag was chromosomally added to the C terminus of *iscR* through splicing by overlap extension (79). The primer pair F*iscR*_cds/R*iscR*_cds (Table 2) was used to amplify ∼500 bp upstream of *iscR* plus the *iscR* coding region excluding the stop codon. The primer pair F3xFLAG/R3xFLAG was used to amplify the 3xFLAG tag. The primer pair F3’*iscR*/R3’*iscR* was used to amplify the ∼500-bp downstream region of *iscR* including the stop codon. These amplified PCR fragments were cloned into a BamHI- and SacI-digested pSR47s suicide plasmid (λ*pir*-dependent replicon, kanamycin resistant [Kan^r^], *sacB* gene conferring sucrose sensitivity) using the NEBuilder HiFi DNA Assembly kit (New England Biolabs, Inc.). Recombinant plasmids were transformed into E. coli S17-1 λ*pir* competent cells and later introduced into Y. pseudotuberculosis IP2666 via conjugation. The resulting Kan^r^, irgansan-resistant (*Yersinia* selective antibiotic) integrants were grown in the absence of antibiotics and plated on sucrose-containing media to select for clones that had lost *sacB* (and by inference, the linked plasmid DNA). Kan^s^, sucrose-resistant, Congo red-positive colonies were screened by PCR and sequenced. *Yersinia* carrying only the 3xFLAG-IscR allele secreted levels of YopE similar to those secreted by the wild-type strain, suggesting that the 3xFLAG tag does not affect the ability of IscR to regulate T3SS gene expression (Fig. S1B). In addition, derepression of *iscS* mRNA was not observed in the 3xFLAG-IscR strain, suggesting the 3xFLAG-IscR retains the ability to repress *iscS* (25) (Fig. S1C). These data demonstrate that the 3xFLAG affinity tag does alter IscR function and thus is suitable for ChIP-Seq experiments.

**TABLE 2:** Y. pseudotuberculosis primers used in this study

Primer	Primer sequence^*a*^	Reference
Fi*scR*_cds	gatatcgaattcctgcagcccggggGCTCCTTAAATTTAGCCATGGC	This work
R*iscR*_cds	ggtctttgtagtcTGCGCGCAGATTGACGTTAATC	This work
F3xFLAG	cgtcaatctgcgcgcaGACTACAAAGACCATGAC	This work
R3xFLAG	ccgcaaattctgcttaCTCGAGTCCACCTTTATC	This work
F3’*iscR*	aggtggactcgagTAAGCAGAATTTGCGGAATTTTAC	This work
R3’*iscR*	ggtggcggccgctctagaactagtgCCTTCAACATAGTTGAAGC	This work
F5’Δ*sufABCDSE*	cgaattcctgcagcccggggTCAATCCTCGTTTTGCGC	This work
R5’Δ*sufABCDSE*	cctccagaccAGAAAATGTCCCAACTGATTC	This work
F5’Δ*DN756_21815-* * DN756_21820*	cgaattcctgcagcccggggGTTGAGTTCTGAAAGAACAATTGTG	This work
R5’Δ*DN756_21815_21820*	ggcgaccttcTTTGCGGTTGGCTTCGATG	This work
F3’Δ*sufABCDSE*	gacattttctGGTCTGGAGGCGATGATC	This work
R3’Δ*sufABCDSE*	agggaacaaaagctggagctATAGTCAGTACTGACCCCG	This work
F3’Δ*DN756_21815-21820*	caaccgcaaaGAAGGTCGCCTCGATAAAG	This work
R3’Δ*DN756_21815_21820*	agggaacaaaagctggagctTTAACTGATCTGCCAGAATTAC	This work
qPCR_*lcrF*_F	GGAGTGATTTTCCGTCAGTA	8
qPCR_*lcrF*_R	CTCCATAAATTTTTGCAACC	8
qPCR_*iscR*_F	CAGGGCGGAAATCGCTGCCT	9
qPCR_*iscR*_R	ATTAGCCGTTGCGGCGCCTAT	9
qPCR_*sufA*_F	CGCAAATTACGCGGCTTATGC	This work
qPCR_*sufA*_R	GGCAGGCTCTTTAGCCATATC	This work
qPCR_*iscS*_F	CGACGCCAGTAGATCCGCGT	19
qPCR_*iscS*_R	ACGAGGGTCTGCACCCACCA	19
qPCR_*yopE*_F	CCATAAACCGGTGGTGAC	98
qPCR_*yopE*_R	CTTGGCATTGAGTGATACTG	98
qPCR_*16s*_F	AGCCAGCGGACCACATAAAG	25
qPCR_*16s*_R	AGTTGCAGACTCCAATCCGG	25
EMSA_*suf*_F	tttttCTCGAGGAGTGTTTTTTCTTTTAGACC	This work
EMSA_*suf*_R	tttttGGATCCTAAACGTTTTCAGGTTGAA	This work
EMSA_*dusBfis*_F	tttttCTCGAGGTATCGATAATATTTCAGTATTAAC	This work
EMSA_*dusBfis*_R	tttttGGATCCGTAGAAATATATCTGTCACACATC	This work
EMSA_*ail*_F	tttttCTCGAGCTGTCACCGTCCTGG	This work
EMSA_*ail*_R	tttttGGATCCGACTAAAGTGGCCAGCC	This work
EMSA_*sodB*_F	tttttCTCGAGTAACTGACGGTCCG	This work
EMSA_*sodB*_R	tttttGGATCCGGTGTGGGGTGAA	This work
EMSA_*katA1*_F	ccgCTCGAGTATTGCTCTCTCATTGTTCGTTATG	This work
EMSA_*katA1*_R	cgcGGATCCATTTGAAATTATGATCATTTGAAATACTGTGC	This work
EMSA_*katA2*_F	ccgCTCGAGGATCGTCAGTTCCCACAG	This work
EMSA_*katA2*_R	cgcGGATCCCCCCACTAGTTGTAGTCG	This work
NdeI-iscR_F	GGAATTCCATATGAGACTGACATCTAAAGGGCGC	This work
BamHI-His6-IscR_R	GCGGGATCCTTAGTGGTGGTGGTGGTGGTGAGCGCGTAACTTAACGTCGATCGC	This work

a Uppercase nucleotides specify primer that anneals to target for molecular cloning; lowercase nucleotides depict complementary sequence for NEB Gibson Assembly or extra nucleotides to facilitate efficient restriction digestion. The XhoI and BamHI restriction sites are underlined.

Generation of the Δ*sufABCDSE* and Δ*DN756_21815-21820* mutants were generated via splicing by overlap extension (79). Primer pairs F5/R5Δ*sufABCDSE* and F5/R5Δ*DN756_21815-21820* (Table 2) were used to amplify ∼1,000 bp 5′ of *sufA* and *DN756_21815*, respectively. Primer pairs F3/R3Δ*sufABCDSE* and F3/R3Δ*DN756_21815-21820* were used to amplify ∼1,000 bp 3′ of *sufE* and *DN756_21820*, respectively. Amplified PCR fragments were cloned into a BamHI- and SacI-digested pSR47s suicide plasmid (λ*pir*-dependent replicon, Kan^r^, *sacB* gene conferring sucrose sensitivity) using the NEBuilder HiFi DNA Assembly kit (New England Biolabs, Inc.). Mutant strains were generated as described above for the 3xFLAG-IscR strain.

### Chromatin immunoprecipitation followed by high-throughput sequencing.

ChIP-Seq experiments were performed in the IscR-3xFLAG strain and the parental wild-type IP2666 strain after growth at 37°C for 3 h in M9+3.6 μM FeSO_4_, using the IP2666plB1 genome (GenBank accession no. CP032566.1 and CP032567.1) for analysis. ChIP assays were performed as previously described (80) using a monoclonal mouse anti-FLAG antibody (Sigma-Aldrich) that enriched for IscR-3XFLAG. Immunoprecipitated DNA was sheared to 200 to 500 bp via probe-based sonication. DNA quantification was carried out using both Agilent High Sensitivity DNA kit and Invitrogen Qubit dsDNA HS assay kit once DNA was immunoprecipitated and purified. For ChIP-Seq experiments, 10 ng of immunoprecipitated and purified DNA fragments from IscR-3XFLAG (three biological replicates) and WT non-FLAG-tagged IscR (one biological replicate), along with 10 ng of input control, were used to generate libraries using Illumina TruSeq ChIP Library Preparation kit. This kit was used following the manufacturer’s instructions, except that the purification of the ligation products was performed using an Invitrogen E-Gel Power Snap system with Invitrogen E-Gel Size Select II 2% agarose gels. Final library validation was performed using the Qubit and Bioanalyzer. This resulted in eight libraries for sequencing: triplicate immunoprecipitated samples and a single negative-control sample, each with a paired input sample. Libraries were sequenced by the University of Wisconsin at Madison Biotechnology Center DNA Sequencing Facility on an Illumina HiSeq to produce 51-bp single-end reads.

Initial quality control checks were performed on FASTQ files with FastQC v0.11.5 (81). Trimmomatic v0.36 (82) was used to remove low-quality reads from the FATSQ files and to also remove Illumina adapters. The reference files for the Y. pseudotuberculosis IP26666pIB1 chromosome and plasmid were downloaded from the NCBI (GenBank accession no. CP032566.1 and CP032567.1) and concatenated into a single file. The trimmed FASTQ files were then aligned to the Y. pseudotuberculosis genome using Bowtie2 v2.3.3.1 (83) with the default settings to generate SAM files. To remove all unaligned reads and all reads which aligned ambiguously, the SAM files were filtered with Samtools v1.6 (84) with the flags “view -q 10.”

Sequences associated with IscR enrichment following precipitation, or “peaks,” were identified using three separate programs: QuEST v2.4 (85), MOSAiCS v2.18.0 (86), and MACS2 (87). QuEST was run according to the manual with the following options: perform false-discovery rate (FDR) analysis, search for punctate peaks (option 1), and use relaxed peak calling criteria (option 3). QuEST was run separately for each of the three immunoprecipitated samples and the negative-control sample using the paired input sample as a background file. From the generated output, the “max_pos” of each identified enriched region was used at the peak summit. Wiggle files were outputted as a default function of QuEST. MOSAiCS was run as a two-sample analysis as described in the vignette (2 May 2019 version) chapters 3 and 5.1 with the same sample pairs as used with QuEST. During the use of MOSAiCS, a fragment and bin size of 175 nucleotides was used. This number was derived by multiplying the average peak shift, as calculated by QuEST from the immunoprecipitated samples, by two. Peak summits and wiggle files were outputted by MOSAiCS as the last steps of the analysis. MACS2 was run according to the manual with a criterion cutoff of FDR value of <E−5, *P* value of <−log_1070_.

Sequences with IscR enrichment following immunoprecipitation were considered *bona fide* ChIP-Seq peaks if, for each peak-calling program, a peak was called in at least two of three samples. Peaks were considered to be the same if their summits fell within 175 bp of each other. At every location where a negative-control peak overlapped an immunoprecipitated sample peak, we manually examined the negative-control data to determine whether the peak should be removed from our analysis. Specifically, we visually looked for negative peaks which were centered on the immunoprecipitated sample peaks and also confirmed that the negative peaks were composed of both forward and reverse mapping reads. MochiView (88) and Tablet (89) were used to visualize the Wig files and binary SAM (BAM) files, respectively. Peaks confirmed to be arising from background noise were removed from our analysis. The average position of the immunoprecipitated sample peak summits was used as the new peak summit moving forward. This process was repeated for each peak-calling program independently, thus yielding three lists of confirmed peak summits. Finally, confirmed peak summits were clustered as described above. All peaks that were identified by at least two of three peak-calling programs were marked as *bona fide* peaks, and other peaks were discarded. Each peak in the final list was examined by eye with MochiView, and particular care was taken to confirm the validity of peaks called by only two of three algorithms. For visualization and graphing purposes, BAM files were displayed on tracks with Integrative Genomics Viewer using BAMCoverage from DeepTools2 (90). Of the 295 peaks, 80% were called by all three peak-calling programs.

### RNA isolation and RNA-Seq.

For RNA isolated from samples subjected to iron starvation, Y. pseudotuberculosis WT and Δ*iscR* strains were grown in 5 ml of M9+3.6 μM FeSO_4_ overnight. Cultures were then diluted to an OD_600_ of 0.1 in 20 ml of M9 medium lacking iron treated with Chelex (Bio-Rad) to remove trace amounts of iron and allowed to grow for 8 h at 26°C (12). The cultures were then diluted again to an OD_600_ of 0.1 into 20-ml Chelex-treated M9 medium and allowed to grow for 12 h at 26˚C. Cultures were then diluted to an OD_600_ of 0.1 into 20-ml Chelex-treated M9 medium with either no iron, 5 μM hemin, or 3.6 μM FeSO_4_. Stock hemin solutions were Chelex treated overnight. After 3 h of growth at 37°C, 5 ml of culture from each condition was pelleted by centrifugation for 5 min at 4,000 rpm. The supernatant was removed, and pellets were resuspended in 500 μl media and treated with 1 ml Bacterial RNA Protect reagent (Qiagen) according to the manufacturer’s protocol. Total RNA was isolated using the RNeasy minikit (Qiagen) per the manufacturer’s protocol. Contaminating DNA was removed using the TURBO DNA-free kit (Life Technologies/Thermo Fischer). rRNA was removed using the RiboMinus Transcriptome isolation kit bacteria (Invitrogen). The cDNA library was prepared using the NEB Ultra Directional RNA Library Prep kit for Illumina. The quality of RNA and cDNA libraries was assessed using an Agilent 2000 Bioanalyzer. Libraries were sequenced using the HiSeq2500 Illumina sequencing platform for 50-bp single reads (University of California [UC] Davis Genome Center).

Trimmomatic (82) version 0.36 was used to trim off low-quality bases and adapter sequences. Trimmed reads were then aligned to a concatenated chromosome (GenBank accession no. CP032566.1) and virulence plasmid (CP032567.1) through Bowtie2 (83). Low-quality aligned reads were removed by filtering out mapped reads with a mapQC score of <10. The BAM file with the aligned reads with a mapQC score of >10 was then split to separate chromosomal mapped reads from virulence plasmid mapped reads. Mapped reads were then normalized to trimmed mean of M-values (TMM), and differential expression analysis was performed using EdgeR (91). Genes were called differentially expressed if the log_2_ fold change (FC) was ≥1 or ≤−1 with an FDR value of <0.01.

### Motif identification and *in silico* search.

IscR binding motif analyses were carried out using the MEME tool from the MEME software suite with default settings (41). The E. coli IscR type I and type II binding motifs were compiled from a training set consisting of known IscR binding sites in E. coli (25, 28). The FIMO tool from the MEME software suite was then used to search for an IscR type I binding motif upstream of *Yersinia iscRSUA*, *cysE*, *erpA*, and *nfuA*.

In order to identify a *Yersinia* IscR consensus binding site from sequences enriched during IscR-FLAG immunoprecipitation, 50 nucleotides within the center of all 176 enriched DNA regions were extracted, and the MEME suite was used to generate a *Yersinia* IscR type II motif. The CentiMo tool from the MEME suite was utilized to assess whether the predicted IscR type II consensus motif was often found near the center of each IscR ChIP-Seq peak.

### Western blot analysis.

Bacterial pellets were resuspended in final sample buffer plus 0.2 M dithiothreitol (FSBS+DTT) and boiled for 15 min. At the time of loading, samples were normalized to the same number of cells by OD_600_. Protein samples were run on a 12.5% sodium dodecyl sulfate-polyacrylamide gel (SDS-PAG) and transferred to a blotting membrane (Immobilon-P) with a wet mini trans-blot cell (Bio-Rad). Blots were blocked for an hour in Tris-buffered saline with Tween 20 and 5% skim milk and probed with rabbit anti-IscR (28), rabbit anti-YopD (gift from Alison Davis and Joan Mecsas), goat anti-YopE (Santa Cruz Biotechnology), rabbit anti-RpoA (gift from Melanie Marketon), and horseradish peroxidase-conjugated secondary antibodies (Santa Cruz Biotech). Gels were imaged by Image Lab software (Bio-Rad).

### Type III secretion assay under non-iron-starved conditions.

Visualization of T3SS cargo secreted in broth culture was performed as previously described (92). For standard Y. pseudotuberculosis T3SS induction (Fig. S1B and Fig. S4C), bacteria were grown overnight in LB medium, subcultured in LB plus 20 mM sodium oxalate (to chelate calcium and induce type III secretion) and 20 mM MgCl_2_ to an OD_600_ of 0.2, grown at 26°C for 1.5 h followed by 37°C for another 1.5 h. Cultures were normalized by OD_600_ and pelleted at 13,200 rpm for 10 min at room temperature. Supernatants were removed, and proteins were precipitated by addition of trichloroacetic acid (TCA) to a final concentration of 10%. Samples were incubated on ice for 20 min and pelleted at 13,200 rpm for 15 min at 4°C. Resulting pellets were washed twice with ice-cold 100% acetone and subsequently resuspended in FSB+DTT. Samples were boiled for 5 min prior to running on a 12.5% SDS-PAG.

### Type III secretion assay under iron-starved aerobic or anaerobic conditions.

We previously found that small amounts of iron are needed to obtain sufficient *Yersinia* growth under anaerobic conditions to detect T3SS activity (9); therefore, we added 0.036 μM to the “low iron” samples for these experiments rather than continuing to iron starve them. Cultures were grown aerobically in Chelex-treated M9 minimal medium plus 0.9% glucose in acid-washed glassware, as previously described (9). Specifically, iron-replete overnight cultures (M9+3.6 μM FeSO_4_) grown at 26°C aerobically were subcultured to an OD_600_ of 0.1 into Chelex-treated M9 medium plus 0.9% glucose and grown for 8 h at 26°C aerobically with agitation. Cultures were then subcultured a second time to OD_600_ 0.1 in fresh Chelex-treated M9 media plus 0.9% glucose and grown for 12 h at 26°C with agitation. Subsequently, cultures were then subcultured a third time to an OD_600_ of 0.2 in M9 medium plus 0.9% glucose supplemented with 3.6 μM FeSO_4_ (iron replete) or with 0.036 μM FeSO_4_ (iron limitation), grown for 2 h at 26°C with agitation, and then shifted to 37°C for 4 h with agitation to induce type III secretion. For anaerobic cultures, the cultures were instead diluted a second time to an OD_600_ of 0.1 in M9 medium plus 0.9% glucose supplemented with 3.6 μM FeSO_4_ (iron replete) or with 0.036 μM FeSO_4_ (iron limitation), and transferred to a vinyl anaerobic chamber where they were grown at 26°C for 12 h. Cultures were then shifted to 37°C for another 4 h to induce type III secretion. Samples were then processed as described above for standard secretion assay conditions.

### Quantitative PCR (qPCR) analysis.

A total of 5 ml of culture from each condition were pelleted by centrifugation for 5 min at 4,000 rpm (4 krpm). The supernatant was removed, and pellets were resuspended in 500 μl of media and treated with 1 ml Bacterial RNA Protect reagent (Qiagen) according to the manufacturer’s protocol. Total RNA was isolated using the RNeasy minikit (Qiagen) per the manufacturer’s protocol. After harvesting total RNA, genomic DNA was removed via the TURBO-DNA-free kit (Life Technologies/Thermo Fisher). cDNA was generated for each sample by using the Moloney murine leukemia virus (M-MLV) reverse transcriptase (Invitrogen) according to the manufacturer’s instructions, as we previously described (19). Power SYBR green PCR master mix (Thermo Fisher Scientific) and primers (Table 2) with optimized concentrations were used to measure target gene levels. The expression levels of each target gene were normalized to that of 16S rRNA present in each sample and calculated by the ΔΔ*C_T_* method. Three independent biological replicates were collected for each tested condition. For each target transcript, significant differential expression between different bacterial strains was defined by *P* value of <0.05 of two-way analysis of variance (ANOVA) (one-way ANOVA with Tukey posttest).

### Cluster of Orthologous Groups (COG) analysis.

The GenBank file of the Y. pseudotuberculosis chromosome (GenBank accession no. CP032566.1) and virulence plasmid (CP032567.1) were downloaded from NCBI. These files were opened using the Artemis Genome Browser (93), where the proteome was exported to a FASTA file. EggNOG 4.5.1 (94) was used to determine the following gene information based on ortholog databases: preferred gene name, COG category, and gene function.

### Cluster analysis.

Cluster analysis was used to cluster gene expression data from the RNA-Seq experiments. The elbow method was first used to determine the appropriate number of clusters. Gene expression data were inputted as normalized reads (TMM) values. These gene expression values were then scaled by gene. R-package pheatmap was used to create clusters dependent on Euclidean distances and a complete method.

### Protein purification and electrophoretic mobility shift assays (EMSAs).

The *iscR* coding sequence was PCR amplified from the E. coli K-12 MG1655 chromosome using primers that incorporated a NdeI restriction site at the 5′ end of the gene, and a BamHI site and His_6_ tag (order listed in 5′–3′ direction) at the 3′ end. The NdeI- and BamHI-digested fragment was cloned into pET11a to generate pPK14263, which was subsequently transformed into strain PK7878 (28). IscR-C92A-His_6_ was purified as previously described for untagged IscR (26, 28). DNA fragments containing the predicted IscR binding region for *ail* (−386 to +14 bp relative to the +1 transcription start site [TSS]), *dusB-fis* (−447 to −247 bp relative to the +1 TSS), *sodB* (−116 to +84 bp relative to the +1 TSS), *katA1* (−240 to −93 bp relative to the +1 TSS), *katA2* (−92 to +1 bp relative to the +1 TSS), and *sufA* (−144 to +56 bp relative to the +1 TSS) were amplified from Y. pseudotuberculosis genomic DNA using primers (Table 2). Amplified products were digested with XhoI and BamHI and subsequently ligated into the pPK7179 plasmid. DNA templates for EMSAs were isolated from plasmid DNA after restriction digestion with XhoI and BamHI. These fragments and linearized plasmid (which served as competitor DNA in the EMSAs) were purified using the QIAquick PCR purification kit (Qiagen). IscR-C92A was incubated with DNA fragments (∼5 to 10 nM) for 30 min at 37°C in 40 mM Tris (pH 7.9), 30 mM KCl, 100 μg/ml bovine serum albumin (BSA), and 1 mM DTT. Glycerol was added to 10%, and samples were loaded onto a nondenaturing 6% polyacrylamide gel in 0.5× Tris-borate-EDTA (TBE) buffer and run at 200 V for 3.5 h. The gel was stained with SYBR green EMSA nucleic acid gel stain (Molecular Probes) and visualized using a Typhoon FLA 900 imager (GE).

### Comparative genomics.

Orthologs of the 213 IscR targets were determined in Y. pseudotuberculosis IP32953 (NC_006155.1), Y. pestis CO92 (NC_003143.1), and Y. enterocolitica 8081 (NC_008800.1) through a combination of BLAST and Mauve, a multiple genome alignment tool (95). Paralogs were avoided by using Mauve. For all orthologs, 1,000 nucleotides upstream and downstream of the start codon were extracted. An IscR binding site was predicted using MEME suite tools and was compared to the known IscR binding site in Y. pseudotuberculosis IP2666. The orthologous IscR binding site was identified based on sequence similarity and distance of the IscR binding site from the start codon. Sequence similarity was calculated by computing the absolute distance between logMEME-FIMO-pvalue in Y. pseudotuberculosis IP2666 (known IscR binding site) compared to logMEME-FIMO-pvalue of orthologous binding site in the indicated strain of *Yersinia* (binding site in question).

### Motility assay.

A total of 1-μl Y. pseudotuberculosis overnight culture was spotted onto motility medium containing 1% tryptone/0.25% agar. The plates were incubated at 26°C for 24 h or 48 h before the diameter of the motile colony was measured.

### Microbial genome browser.

Both ChIP-Seq and RNA-Seq tracks were deposited to the UCSC Microbial Genome browser. For RNA-Seq data, final BAM file alignments were converted to bigwig files using bamCoverage-deepTools (90). Chromosomal alignments were normalized by counts per million mapped reads (CPM), while alignments to the virulence plasmid were not normalized due to the known increase in plasmid copy number at 37°C relative to the chromosome (96). For ChIP-Seq data, both BAM files that were mapped to the chromosome or virulence plasmid were normalized by CPM. The RNA-Seq and ChIP-Seq data are available for viewing, and the track hub data can be found at http://zam.soe.ucsc.edu/hubs/StoneYersinia/hub.txt.
