# Supplementary material for: Genome Scale Analysis Reveals IscR Directly and Indirectly Regulates Virulence Factor Genes in Pathogenic Yersinia
Source: mBio. 2021 Jun 1;12(3):e00633-21. doi: 10.1128/mBio.00633-21 (PMC8262890; doi:10.1128/mBio.00633-21)
Supplement: FIG S1 [file mbio.00633-21-sf001.pdf]

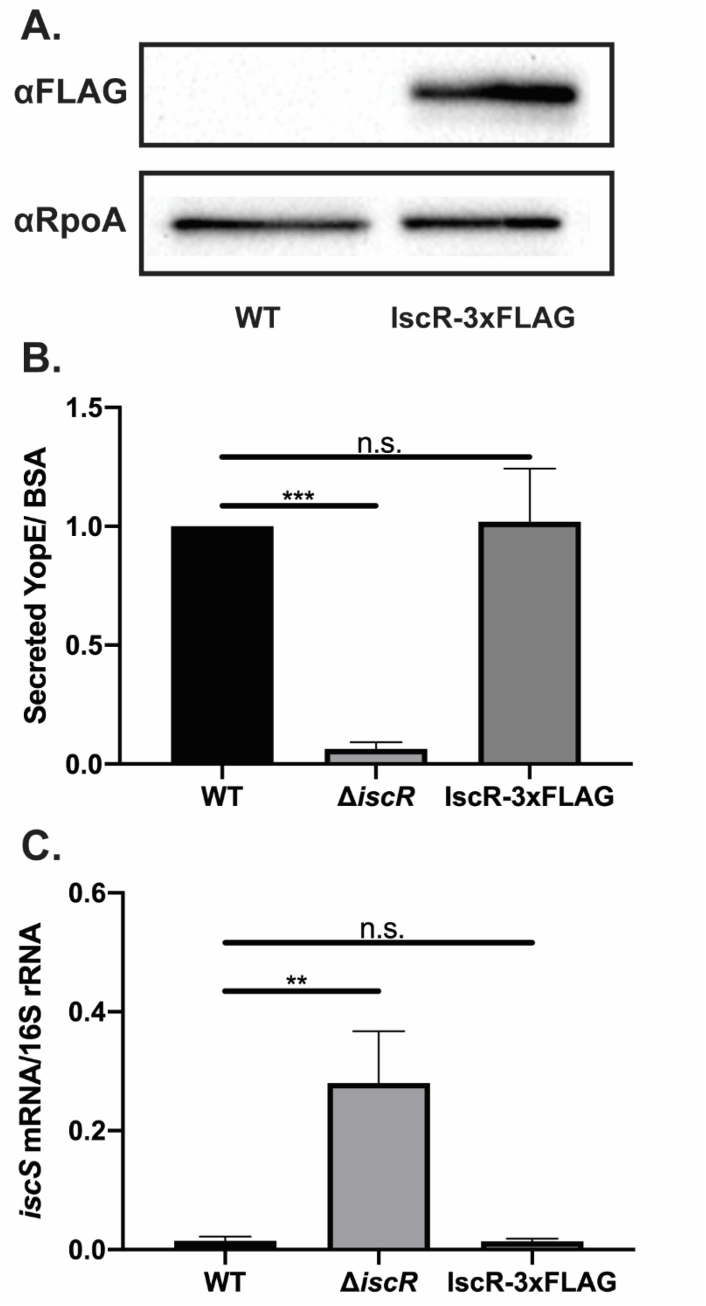

Figure S1. 3xFLAG-tagged IscR rescues an *iscR* deletion. (A) Whole cell extracts from WT *Y. pseudotuberculosis* or a strain harboring a chromosomally-encoded 3xFLAG tagged IscR were visualized using anti-FLAG or anti-RpoA antibodies. **(B)** To measure the relative efficiency of the Ysc T3SS, *Yersinia* strains were grown under T3SS-inducing conditions and secreted proteins precipitated by trichloroacetic acid were visualized using Coomassie blue. Relative amounts of the T3SS effector protein YopE were quantified by densitometry compared to a spiked in BSA protein control. The average of three biological replicates ± standard deviation is shown. **(C)** *Yersinia* strains were grown under T3SS inducing conditions and relative *iscS* mRNA levels evaluated by qPCR and normalized to 16s rRNA. The average of three biological replicates ± standard deviation is shown. ***p<0.001; **p<0.01; n.s. non-significant (one-way ANOVA with Dunnett's *post-hoc* test).
