# Supplementary material for: Genome Scale Analysis Reveals IscR Directly and Indirectly Regulates Virulence Factor Genes in Pathogenic Yersinia
Source: mBio. 2021 Jun 1;12(3):e00633-21. doi: 10.1128/mBio.00633-21 (PMC8262890; doi:10.1128/mBio.00633-21)
Supplement: FIG S2 [file mbio.00633-21-sf002.pdf]

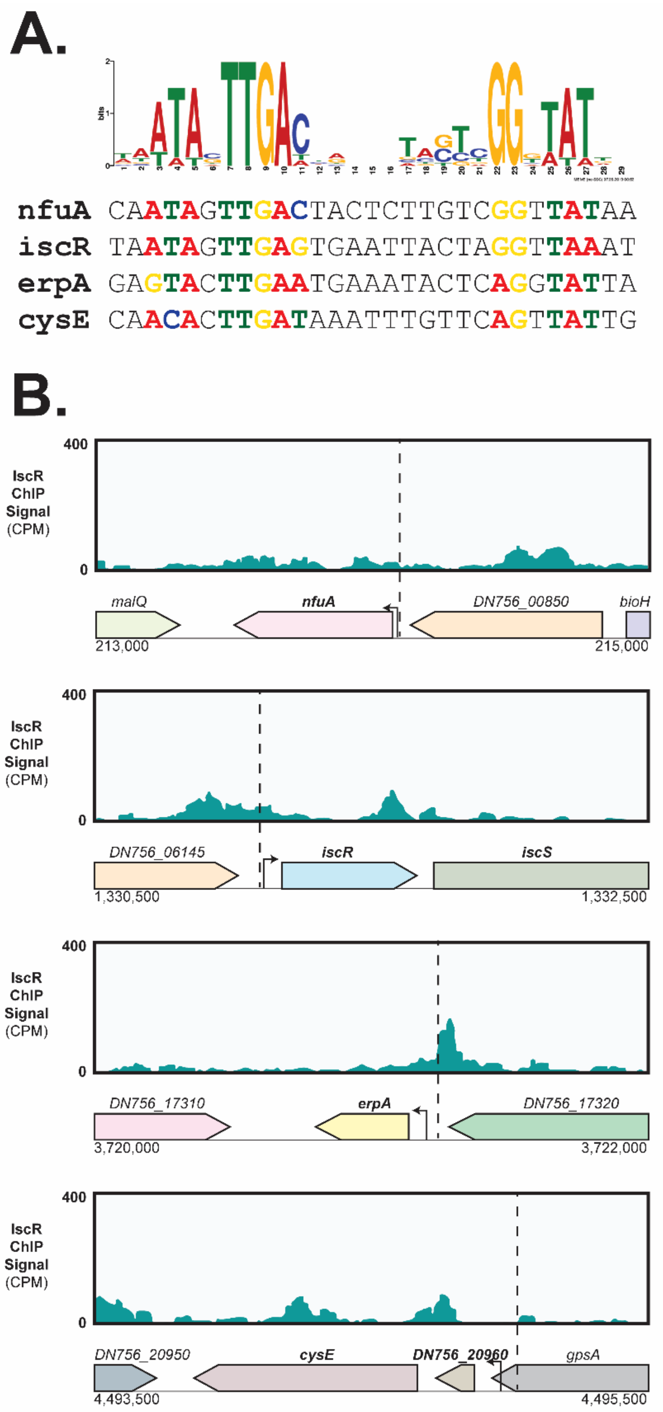

Figure S2. Global IscR ChIP-seq analysis fails to identify predicted IscR Type I sites. (A) Known *E. coli* IscR type I binding sites were used to generate a IscR type I binding motif using MEME-suite tools. The *Y. pseudotuberculosis* IP2666 genome was scanned for IscR type I sites using FIMO specifically upstream of the *nfuA, iscRSUA, erpA, and DN756_20960_cysE* promoters. The predicted sequences were aligned to the *E. coli* consensus IscR type I binding motif. **(B)** IscR ChIP-seq plots illustrating read coverage of IscR-binding peaks assigned to the promoter of *nfuA*, *iscRSUA*, *erpA*, and *DN756_20960_cysE* from all three replicates combined. Dashed lines correspond to the zenith of the predicted 29 bp IscR type I motifs.
