# Supplementary material for: Genome Scale Analysis Reveals IscR Directly and Indirectly Regulates Virulence Factor Genes in Pathogenic Yersinia
Source: mBio. 2021 Jun 1;12(3):e00633-21. doi: 10.1128/mBio.00633-21 (PMC8262890; doi:10.1128/mBio.00633-21)
Supplement: FIG S3 [file mbio.00633-21-sf003.pdf]

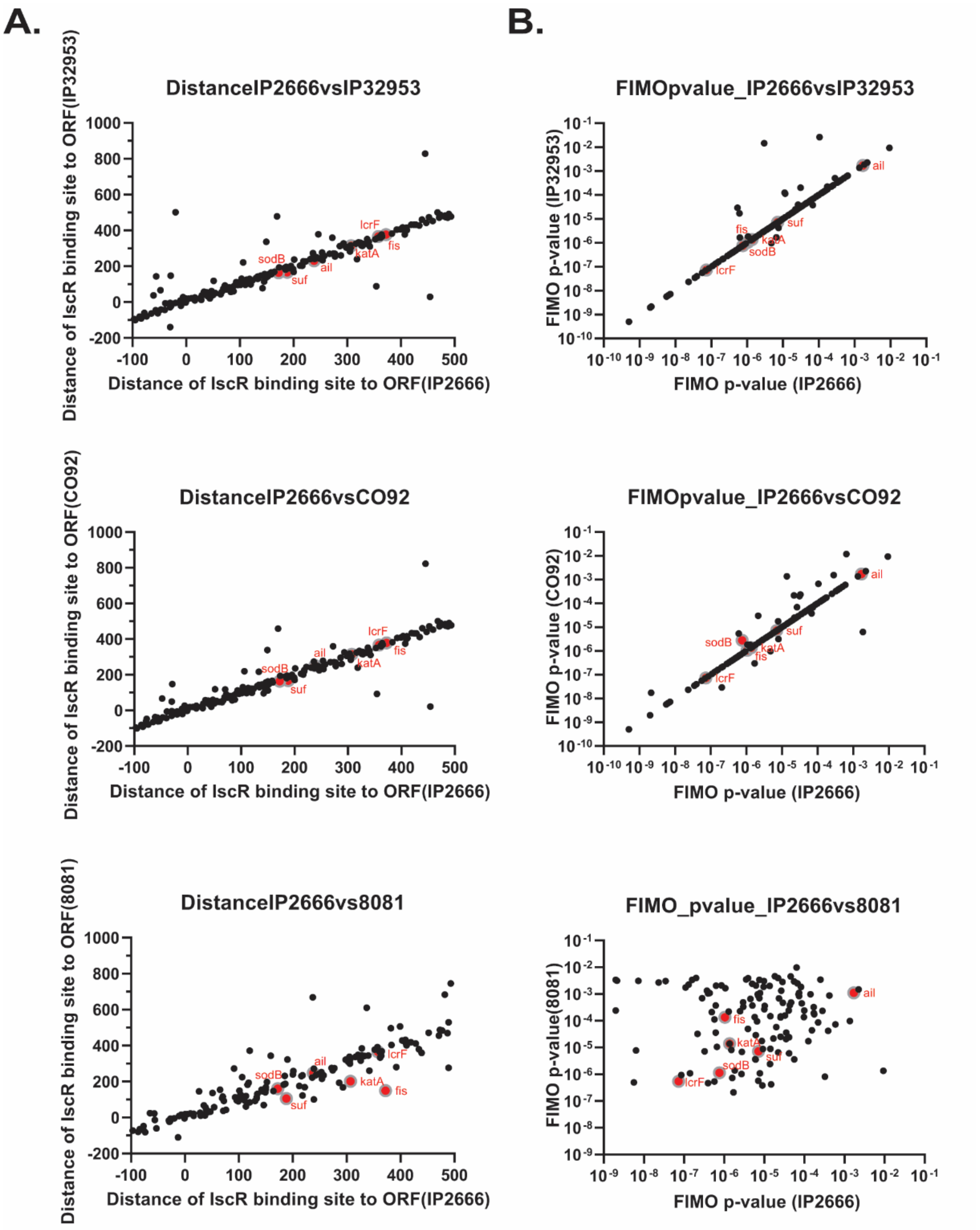

Figure S3. Conservation of IscR binding sites in *Y. pseudotuberculosis* (IP2666, IP32953), *Y. pestis* (CO92), and *Y. enterocolitica* (8081). (A) Distance of the identified IscR binding site in *Y. pseudotuberculosis* (IP2666) from the start codon of each transcription unit member of the IscR regulon is plotted versus the distance of the predicted IscR binding site from the start codon of the identified ortholog in *Y. pseudotuberculosis* (IP32953), *Y. pestis* (CO92), or *Y. enterocolitica* (8081). **(B)** The $\log_{10}$ FIMO p-value of the identified IscR binding site in *Y. pseudotuberculosis* (IP2666) is plotted versus the $\log_{10}$ FIMO p-value of the predicted IscR binding site upstream of identified orthologs in *Y. pseudotuberculosis* (IP32953), *Y. pestis* (CO92), or *Y. enterocolitica* (8081).
