# Supplementary material for: Genome Scale Analysis Reveals IscR Directly and Indirectly Regulates Virulence Factor Genes in Pathogenic Yersinia
Source: mBio. 2021 Jun 1;12(3):e00633-21. doi: 10.1128/mBio.00633-21 (PMC8262890; doi:10.1128/mBio.00633-21)
Supplement: FIG S4 [file mbio.00633-21-sf004.pdf]

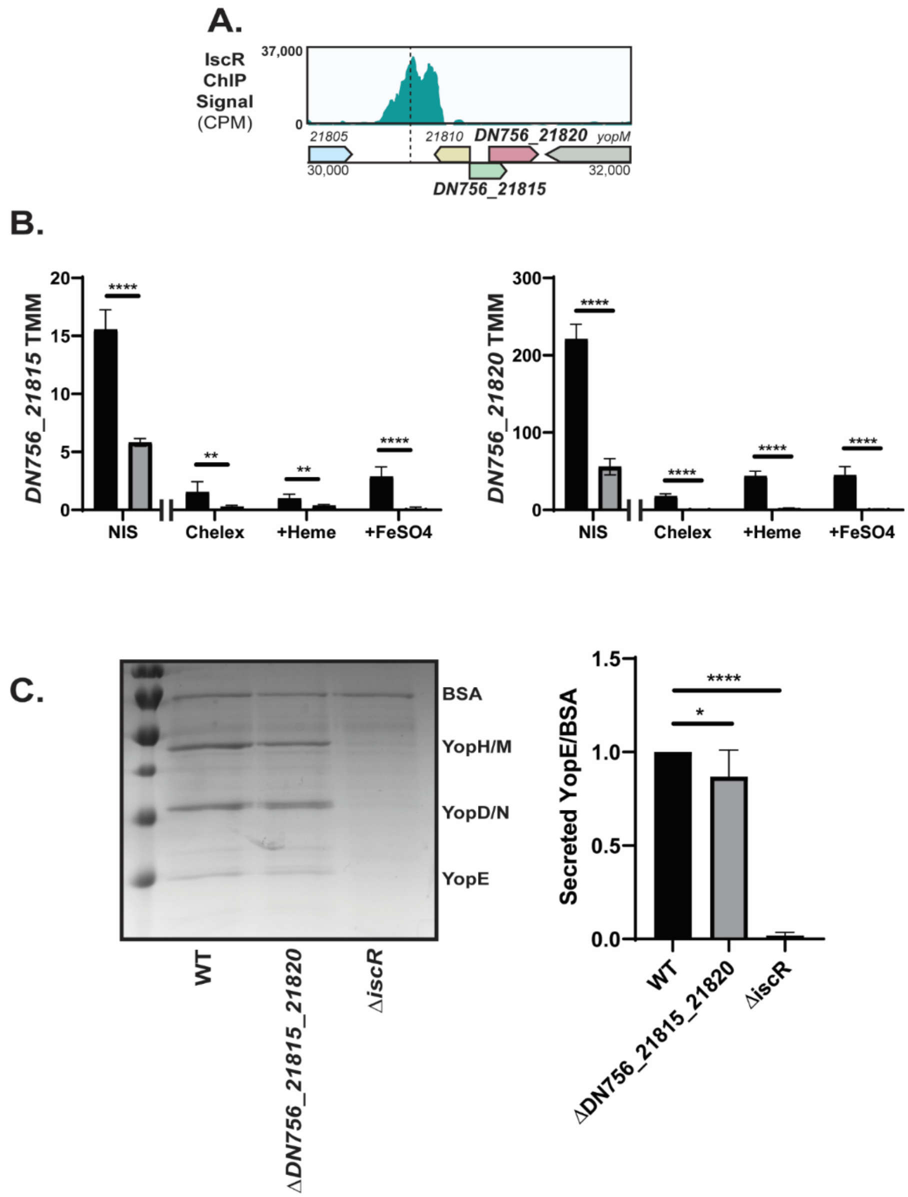


**Figure S4. Deletion of DN756_21815 and DN756_21820, identified gene targets of IscR, results in a small but significant decrease in type III secretion. (A)** Read coverage of IscR-binding peaks proximal to *DN756_21815_DN756_21820*. Counts per million reads (CPM) are plotted versus the genomic position. Dashed lines correspond to the predicted 30 bp IscR type II motif. Arrows indicate transcriptional start sites (50). **(B)** Expression of *DN756_21815*, and *DN756_21820* genes under varying iron conditions as measured by RNA-seq. Reads are represented by Trimmed Mean of M-values (TMM) of WT (black) and Δ*iscR* strains (grey) grown in M9 minimal media containing $FeSO_4$ (non-iron starved, NIS), iron starved in chelex-treated M9 minimal media with no iron source added back (Chelex), supplemented with 5 mM hemin (+Heme), or supplemented with $FeSO_4$ (+$FeSO_4$).**p<0.01; ***p<0.001; ****p<0.0001 (EdgeR with a corrected FDR post-hoc test). **(C)** *Yersinia* strains were grown under rich media, T3SS inducing conditions. The secretome of these cultures was visualized with Coomassie blue. The effector protein, YopE, was quantified by densitometry relative to the WT control to measure the relative efficiency of the Ysc T3SS. The average of 5 biological replicates ± standard deviation is shown. ****p<0.0001; *p<0.05 (one way ANOVA with Dunnett's post-hoc test).
