# Supplementary material for: Genome Scale Analysis Reveals IscR Directly and Indirectly Regulates Virulence Factor Genes in Pathogenic Yersinia
Source: mBio. 2021 Jun 1;12(3):e00633-21. doi: 10.1128/mBio.00633-21 (PMC8262890; doi:10.1128/mBio.00633-21)
Supplement: FIG S5 [file mbio.00633-21-sf005.pdf]

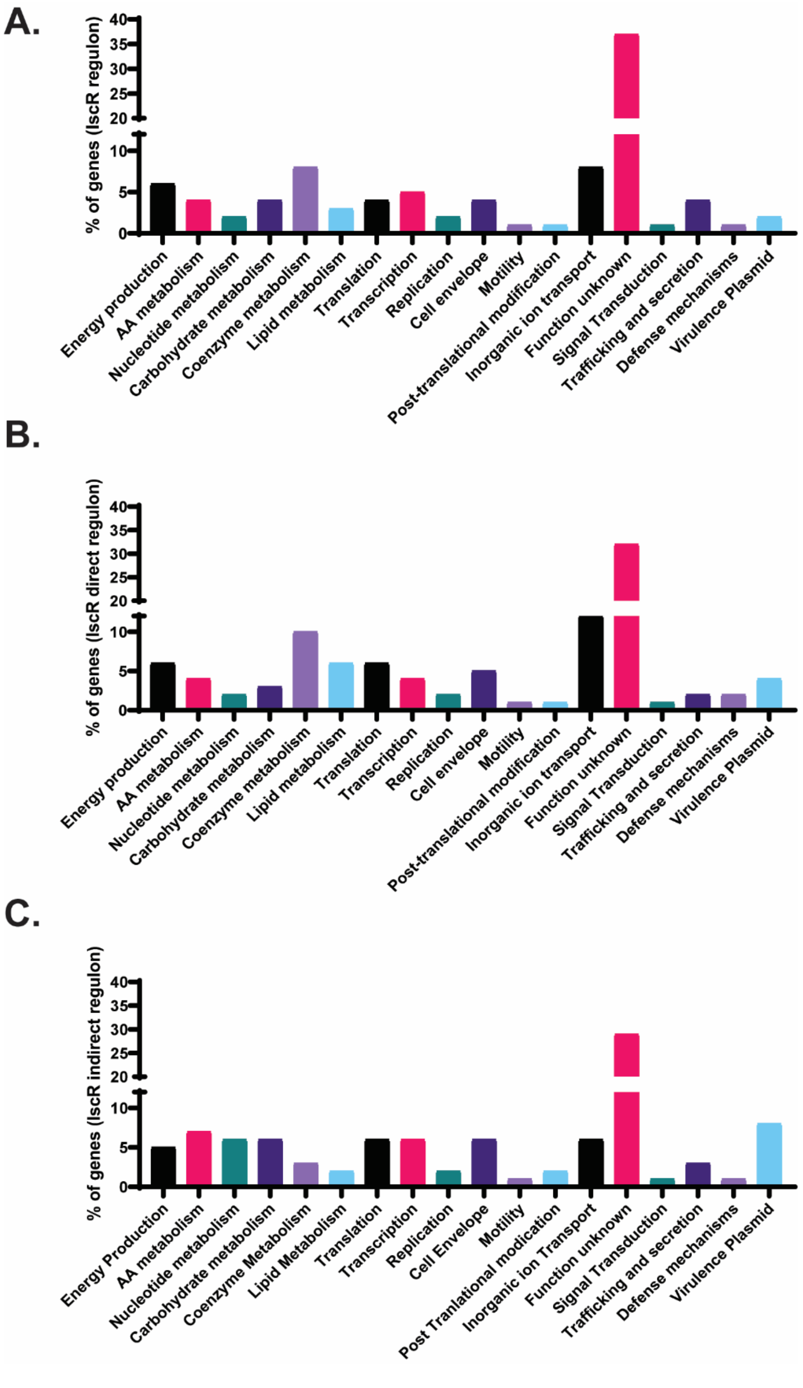

Figure S5. COG analysis of the IscR regulon, functional regulon, and indirect regulon. Clusters of Orthologous Groups of proteins (COG) analysis of genes with a IscR ChIP-Seq peak **(A)**, genes with a IscR ChIP-Seq peak and are differentially expressed between WT and the *iscR* mutant under at least one tested condition **(B)**, and genes differentially expressed between WT and the iscR mutant but have no IscR ChIP-Seq peak **(C)**.
