# Supplementary material for: Genome Scale Analysis Reveals IscR Directly and Indirectly Regulates Virulence Factor Genes in Pathogenic Yersinia
Source: mBio. 2021 Jun 1;12(3):e00633-21. doi: 10.1128/mBio.00633-21 (PMC8262890; doi:10.1128/mBio.00633-21)
Supplement: FIG S6 [file mbio.00633-21-sf006.pdf]

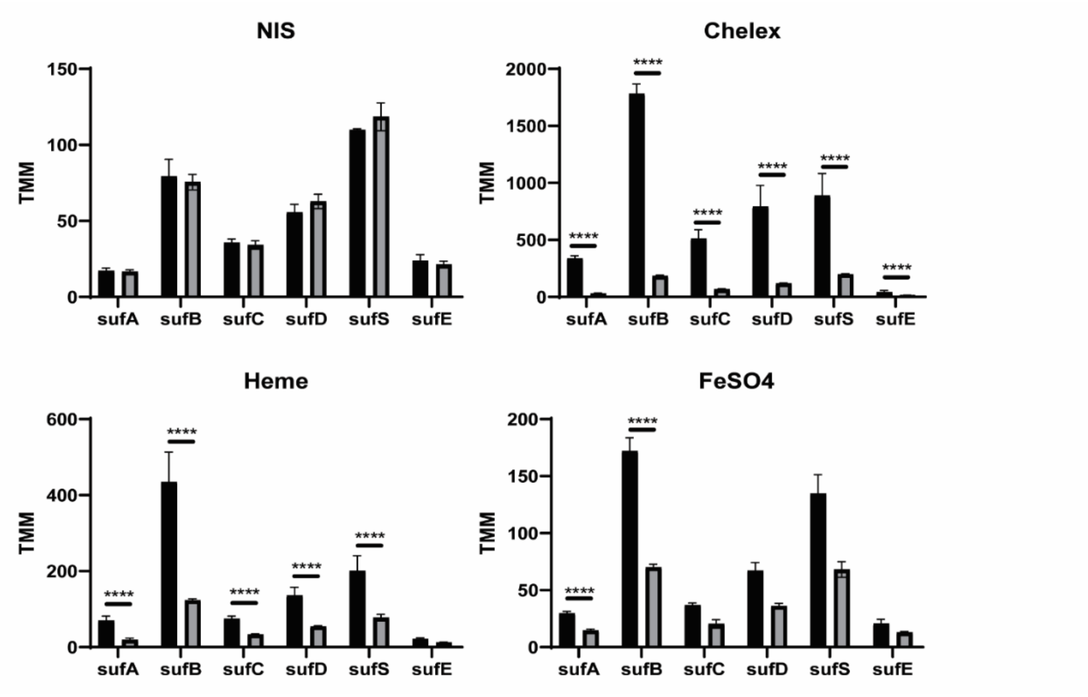

Figure S6. Expression of the *suf* operon is modulated by iron and requires IscR . Expression of *sufA*, *sufB*, *sufC*, *sufD*, *sufS*, and *sufE* genes under varying iron conditions as measured by RNA-seq. Reads are represented by Trimmed Mean of M-values (TMM) of WT (black) and Δ*iscR* strains (grey) grown in M9 minimal media containing 3.6 µM $FeSO_4$ (non-iron starved, NIS), iron starved in chelex-treated M9 minimal media with no iron source added back (Chelex), supplemented with 5 mM hemin (+Heme), or supplemented with 3.6 µM $FeSO_4$ (+$FeSO_4$).**p<0.01; ***p<0.001; ****p<0.0001 (EdgeR with a corrected FDR post-hoc test).
