# Supplementary material for: Genome Scale Analysis Reveals IscR Directly and Indirectly Regulates Virulence Factor Genes in Pathogenic Yersinia
Source: mBio. 2021 Jun 1;12(3):e00633-21. doi: 10.1128/mBio.00633-21 (PMC8262890; doi:10.1128/mBio.00633-21)
Supplement: FIG S7 [file mbio.00633-21-sf007.pdf]

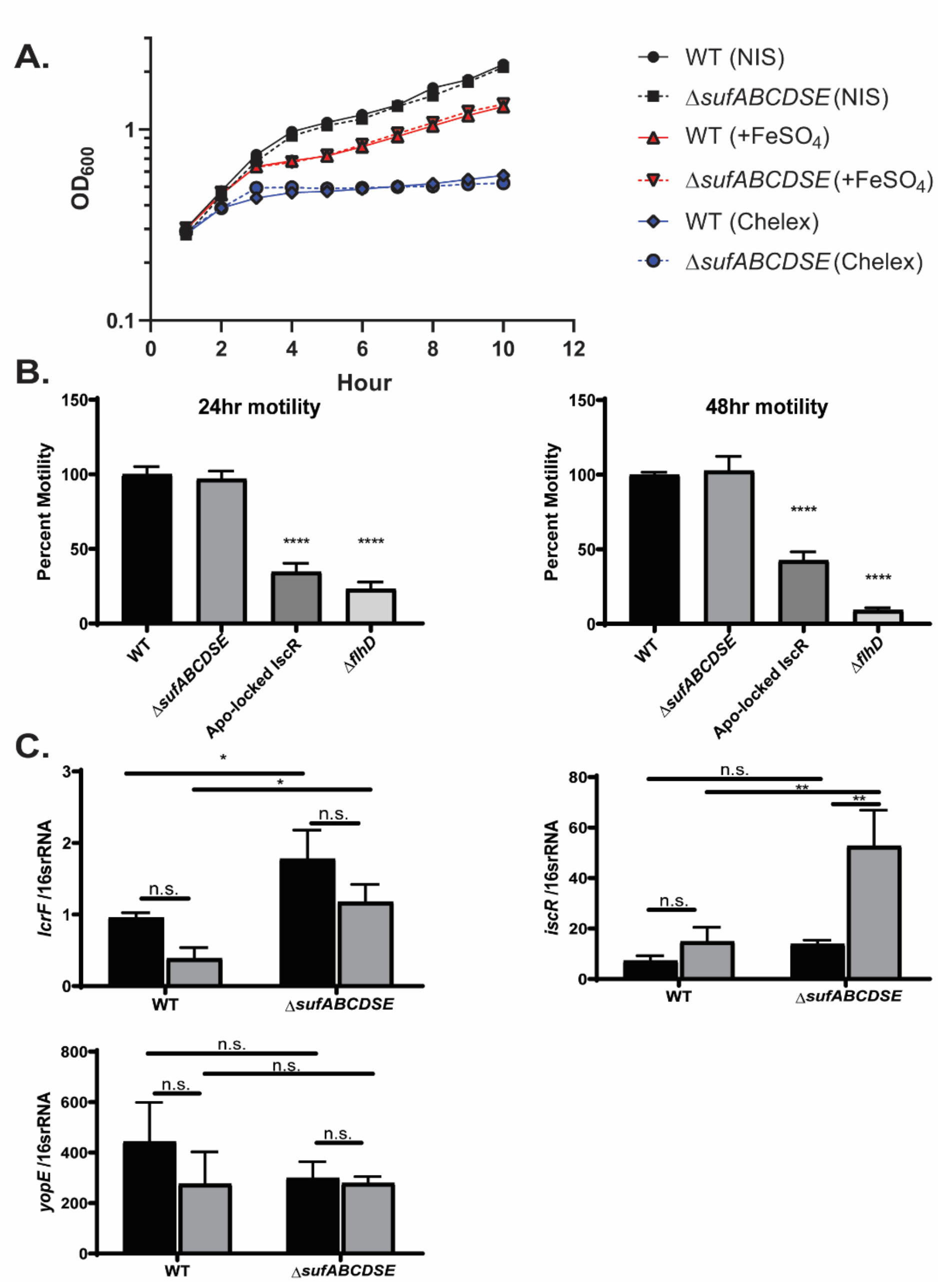

Figure S7. The Suf pathway does not affect motility or growth. (A) Iron starved *Y. pseudotuberculosis* were either kept in iron starved chelex-treated M9 minimal media with no iron source added back (Chelex), or supplemented with 3.6 µM $FeSO_4$ (+$FeSO_4$). Cultures were grown at 26°C for 10 hr and optical density at 600 nm measured every hour. Non-iron starved (NIS) samples were never exposed to chelex-treated M9. **(B)** The indicated strains were spotted onto Motility agar (1% Tryptone 0.25% Agar) and were grown at 26°C. The diameters of the colonies were measured 24 hr and 48 hr later and used to calculate percent motility. Graphs represent three biological replicates. ****$p<0.0001$ (one way ANOVA with Dunnett's post-hoc test). **(C)** Expression of *lcrF*, *iscR*, *yopE*, and *sufA*, relative to 16s rRNA, following iron starved conditions in M9 minimal media at 37˚C, as measured by qPCR. Black bars represent cultures which were iron starved and supplemented with 3.6 µM $FeSO_4$ before induction of the type III secretion system, while grey bars represent cultures that received 0.036 µM $FeSO_4$ before induction of the type III secretion system. Average of three independent experiments ± standard deviation is shown. ***$p<0.001$; **$p<0.01$; *$p<0.05$ (one way ANOVA with Dunnett's post-hoc test).
